# International Consensus Based Review and Recommendations for Minimum Reporting Standards in Research on Transcutaneous Vagus Nerve Stimulation (Version 2020)

**DOI:** 10.3389/fnhum.2020.568051

**Published:** 2021-03-23

**Authors:** Adam D. Farmer, Adam Strzelczyk, Alessandra Finisguerra, Alexander V. Gourine, Alireza Gharabaghi, Alkomiet Hasan, Andreas M. Burger, Andrés M. Jaramillo, Ann Mertens, Arshad Majid, Bart Verkuil, Bashar W. Badran, Carlos Ventura-Bort, Charly Gaul, Christian Beste, Christopher M. Warren, Daniel S. Quintana, Dorothea Hämmerer, Elena Freri, Eleni Frangos, Eleonora Tobaldini, Eugenijus Kaniusas, Felix Rosenow, Fioravante Capone, Fivos Panetsos, Gareth L. Ackland, Gaurav Kaithwas, Georgia H. O'Leary, Hannah Genheimer, Heidi I. L. Jacobs, Ilse Van Diest, Jean Schoenen, Jessica Redgrave, Jiliang Fang, Jim Deuchars, Jozsef C. Széles, Julian F. Thayer, Kaushik More, Kristl Vonck, Laura Steenbergen, Lauro C. Vianna, Lisa M. McTeague, Mareike Ludwig, Maria G. Veldhuizen, Marijke De Couck, Marina Casazza, Marius Keute, Marom Bikson, Marta Andreatta, Martina D'Agostini, Mathias Weymar, Matthew Betts, Matthias Prigge, Michael Kaess, Michael Roden, Michelle Thai, Nathaniel M. Schuster, Nicola Montano, Niels Hansen, Nils B. Kroemer, Peijing Rong, Rico Fischer, Robert H. Howland, Roberta Sclocco, Roberta Sellaro, Ronald G. Garcia, Sebastian Bauer, Sofiya Gancheva, Stavros Stavrakis, Stefan Kampusch, Susan A. Deuchars, Sven Wehner, Sylvain Laborde, Taras Usichenko, Thomas Polak, Tino Zaehle, Uirassu Borges, Vanessa Teckentrup, Vera K. Jandackova, Vitaly Napadow, Julian Koenig

**Affiliations:** ^1^Department of Gastroenterology, University Hospitals of North Midlands NHS Trust, Stoke on Trent, United Kingdom; ^2^Department of Neurology, Epilepsy Center Frankfurt Rhine-Main, Goethe-University Frankfurt, Frankfurt am Main, Germany; ^3^Scientific Institute, IRCCS E. Medea, Pasian di Prato, Italy; ^4^Department of Neuroscience, Physiology and Pharmacology, Centre for Cardiovascular and Metabolic Neuroscience, University College London, London, United Kingdom; ^5^Institute for Neuromodulation and Neurotechnology, University Hospital and University of Tuebingen, Tuebingen, Germany; ^6^Department of Psychiatry, Psychotherapy and Psychosomatics, Medical Faculty, University of Augsburg, Augsburg, Germany; ^7^Department of Psychiatry and Psychotherapy, University Hospital, LMU Munich, Munich, Germany; ^8^Laboratory for Biological Psychology, Faculty of Psychology and Educational Sciences, University of Leuven, Leuven, Belgium; ^9^Leibniz Institute for Neurobiology, Magdeburg, Germany; ^10^Department of Neurology, Institute for Neuroscience, 4Brain, Ghent University Hospital, Gent, Belgium; ^11^Sheffield Institute for Translational Neuroscience (SITraN), University of Sheffield, Sheffield, United Kingdom; ^12^Clinical Psychology and the Leiden Institute of Brain and Cognition, Leiden University, Leiden, Netherlands; ^13^Department of Psychiatry, Medical University of South Carolina, Charleston, SC, United States; ^14^Department of Biological Psychology and Affective Science, Faculty of Human Sciences, University of Potsdam, Potsdam, Germany; ^15^Migraine and Headache Clinic Koenigstein, Königstein im Taunus, Germany; ^16^Cognitive Neurophysiology, Department of Child and Adolescent Psychiatry, Faculty of Medicine, TU Dresden, Dresden, Germany; ^17^Utah State University, Logan, UT, United States; ^18^NORMENT, Division of Mental Health and Addiction, University of Oslo and Oslo University Hospital, Oslo, Norway; ^19^Department of Psychology, University of Oslo, Oslo, Norway; ^20^KG Jebsen Centre for Neurodevelopmental Disorders, University of Oslo, Oslo, Norway; ^21^Medical Faculty, Institute of Cognitive Neurology and Dementia Research, Otto-von-Guericke University, Magdeburg, Germany; ^22^Institute of Cognitive Neuroscience, University College London, London, United Kingdom; ^23^Center for Behavioral Brain Sciences Magdeburg (CBBS), Otto-von-Guericke University, Magdeburg, Germany; ^24^Department of Pediatric Neuroscience, Fondazione IRCCS Istituto Neurologico Carlo Besta, Milan, Italy; ^25^Pain and Integrative Neuroscience Branch, National Center for Complementary and Integrative Health, NIH, Bethesda, MD, United States; ^26^Department of Internal Medicine, Fondazione IRCCS Ca' Granda, Ospedale Maggiore Policlinico, Milan, Italy; ^27^Department of Clinical Sciences and Community Health, University of Milan, Milan, Italy; ^28^Institute of Electrodynamics, Microwave and Circuit Engineering, TU Wien, Vienna, Austria; ^29^SzeleSTIM GmbH, Vienna, Austria; ^30^Unit of Neurology, Neurophysiology, Neurobiology, Department of Medicine, Università Campus Bio-Medico di Roma, Rome, Italy; ^31^Faculty of Biology and Faculty of Optics, Complutense University of Madrid and Institute for Health Research, San Carlos Clinical Hospital (IdISSC), Madrid, Spain; ^32^Translational Medicine and Therapeutics, Barts and The London School of Medicine and Dentistry, William Harvey Research Institute, Queen Mary University of London, London, United Kingdom; ^33^Department of Pharmaceutical Sciences, School of Biosciences and Biotechnology, Babasaheb Bhimrao Ambedkar University (A Central University), Lucknow, India; ^34^Department of Biological Psychology, Clinical Psychology and Psychotherapy, University of Würzburg, Würzburg, Germany; ^35^Division of Nuclear Medicine and Molecular Imaging, Department of Radiology, Massachusetts General Hospital and Harvard Medical School, Boston, MA, United States; ^36^Faculty of Health, Medicine and Life Sciences, School for Mental Health and Neuroscience, Alzheimer Centre Limburg, Maastricht University, Maastricht, Netherlands; ^37^Research Group Health Psychology, Faculty of Psychology and Educational Sciences, University of Leuven, Leuven, Belgium; ^38^Headache Research Unit, Department of Neurology-Citadelle Hospital, University of Liège, Liège, Belgium; ^39^Functional Imaging Lab, Department of Radiology, Guang An Men Hospital, China Academy of Chinese Medical Sciences, Beijing, China; ^40^School of Biomedical Science, Faculty of Biological Science, University of Leeds, Leeds, United Kingdom; ^41^Division for Vascular Surgery, Department of Surgery, Medical University of Vienna, Vienna, Austria; ^42^Department of Psychological Science, University of California, Irvine, Irvine, CA, United States; ^43^Institute for Cognitive Neurology and Dementia Research, Otto-von-Guericke-University Magdeburg, Magdeburg, Germany; ^44^Neuromodulatory Networks, Leibniz Institute for Neurobiology, Magdeburg, Germany; ^45^Clinical and Cognitive Psychology and the Leiden Institute of Brain and Cognition, Leiden University, Leiden, Netherlands; ^46^NeuroV̇ASQ̇ - Integrative Physiology Laboratory, Faculty of Physical Education, University of Brasilia, Brasilia, Brazil; ^47^Department of Anatomy, Faculty of Medicine, Mersin University, Mersin, Turkey; ^48^Mental Health and Wellbeing Research Group, Vrije Universiteit Brussel, Brussels, Belgium; ^49^Faculty of Health Care, University College Odisee, Aalst, Belgium; ^50^Division of Epileptology, Fondazione IRCCS Istituto Neurologico C. Besta, Milan, Italy; ^51^Department of Neurosurgery, University of Tübingen, Tübingen, Germany; ^52^Department of Biomedical Engineering, City College of New York, New York, NY, United States; ^53^Department of Psychology, Education and Child Studies, Erasmus University Rotterdam, Rotterdam, Netherlands; ^54^Faculty of Health Sciences Brandenburg, University of Potsdam, Potsdam, Germany; ^55^Deutsches Zentrum für Neurodegenerative Erkrankungen (DZNE), Magdeburg, Germany; ^56^Center for Behavioral Brain Sciences, Otto-von-Guericke University, Magdeburg, Germany; ^57^University Hospital of Child and Adolescent Psychiatry and Psychotherapy, University of Bern, Bern, Switzerland; ^58^Section for Translational Psychobiology in Child and Adolescent Psychiatry, Department of Child and Adolescent Psychiatry, Centre for Psychosocial Medicine, University of Heidelberg, Heidelberg, Germany; ^59^Division of Endocrinology and Diabetology, Medical Faculty, Heinrich-Heine University Düsseldorf, Düsseldorf, Germany; ^60^Institute for Clinical Diabetology, German Diabetes Center, Leibniz Center for Diabetes Research at Heinrich Heine University, Düsseldorf, Germany; ^61^German Center for Diabetes Research, Munich, Germany; ^62^Department of Psychology, College of Liberal Arts, University of Minnesota, Minneapolis, MN, United States; ^63^Department of Anesthesiology, Center for Pain Medicine, University of California, San Diego Health System, La Jolla, CA, United States; ^64^Department of Psychiatry and Psychotherapy, University of Göttingen, Göttingen, Germany; ^65^Laboratory of Systems Neuroscience and Imaging in Psychiatry (SNIPLab), University of Göttingen, Göttingen, Germany; ^66^Department of Psychiatry and Psychotherapy, University of Tübingen, Tübingen, Germany; ^67^Institute of Acupuncture and Moxibustion, China Academy of Chinese Medical Sciences, Beijing, China; ^68^Department of Psychology, University of Greifswald, Greifswald, Germany; ^69^Department of Psychiatry, University of Pittsburgh School of Medicine, UPMC Western Psychiatric Hospital, Pittsburgh, PA, United States; ^70^Department of Radiology, Athinoula A. Martinos Center for Biomedical Imaging, Massachusetts General Hospital, Charlestown, MA, United States; ^71^Department of Radiology, Logan University, Chesterfield, MO, United States; ^72^Cognitive Psychology Unit, Institute of Psychology, Leiden University, Leiden, Netherlands; ^73^Leiden Institute for Brain and Cognition, Leiden, Netherlands; ^74^Department of Developmental Psychology and Socialisation, University of Padova, Padova, Italy; ^75^Athinoula A. Martinos Center for Biomedical Imaging, Department of Radiology, Massachusetts General Hospital, Harvard Medical School, Charlestown, MA, United States; ^76^Department of Psychiatry, Massachusetts General Hospital, Harvard Medical School, Boston, MA, United States; ^77^Heart Rhythm Institute, University of Oklahoma Health Sciences Center, Oklahoma City, OK, United States; ^78^Faculty of Biological Science, School of Biomedical Science, University of Leeds, Leeds, United Kingdom; ^79^Department of Surgery, University Hospital Bonn, Bonn, Germany; ^80^Department of Performance Psychology, Institute of Psychology, Deutsche Sporthochschule, Köln, Germany; ^81^Department of Anesthesiology, University Medicine Greifswald, Greifswald, Germany; ^82^Department of Anesthesia, McMaster University, Hamilton, ON, Canada; ^83^Laboratory of Functional Neurovascular Diagnostics, AG Early Diagnosis of Dementia, Department of Psychiatry, Psychosomatics and Psychotherapy, University Clinic Würzburg, Würzburg, Germany; ^84^Department of Neurology, Otto-von-Guericke University, Magdeburg, Germany; ^85^Department of Social and Health Psychology, Institute of Psychology, Deutsche Sporthochschule, Köln, Germany; ^86^Department of Epidemiology and Public Health, Faculty of Medicine, University of Ostrava, Ostrava, Czechia; ^87^Department of Human Movement Studies, Faculty of Education, University of Ostrava, Ostrava, Czechia; ^88^Section for Experimental Child and Adolescent Psychiatry, Department of Child and Adolescent Psychiatry, Centre for Psychosocial Medicine, University of Heidelberg, Heidelberg, Germany

**Keywords:** transcutaneous vagus nerve stimulation, minimum reporting standards, guidelines & recommendations, transcutaneous auricular vagus nerve stimulation, transcutaneous cervical vagus nerve stimulation

## Abstract

Given its non-invasive nature, there is increasing interest in the use of transcutaneous vagus nerve stimulation (tVNS) across basic, translational and clinical research. Contemporaneously, tVNS can be achieved by stimulating either the auricular branch or the cervical bundle of the vagus nerve, referred to as transcutaneous auricular vagus nerve stimulation(VNS) and transcutaneous cervical VNS, respectively. In order to advance the field in a systematic manner, studies using these technologies need to adequately report sufficient methodological detail to enable comparison of results between studies, replication of studies, as well as enhancing study participant safety. We systematically reviewed the existing tVNS literature to evaluate current reporting practices. Based on this review, and consensus among participating authors, we propose a set of minimal reporting items to guide future tVNS studies. The suggested items address specific technical aspects of the device and stimulation parameters. We also cover general recommendations including inclusion and exclusion criteria for participants, outcome parameters and the detailed reporting of side effects. Furthermore, we review strategies used to identify the optimal stimulation parameters for a given research setting and summarize ongoing developments in animal research with potential implications for the application of tVNS in humans. Finally, we discuss the potential of tVNS in future research as well as the associated challenges across several disciplines in research and clinical practice.

## Introduction

### Brief History of Transcutaneous Vagus Nerve Stimulation

The vagus nerve (VN) is the Xth cranial nerve and the longest nerve, which courses from the brainstem to the distal third of the colon. It is the main neural substrate of the parasympathetic nervous system and is composed of afferent and efferent pathways, although the former predominate (80%) (Butt et al., 2020). As part of a complex network of neural structures that serves to maintain psychophysiological balance in the organism, its importance cannot be underestimated. The vagus “nerve” is actually two nerves, a left vagus and a right vagus, with slightly different neural origins and targets. It is composed of different types of fibers that vary in myelination, size, and conduction speed (e.g., *for an excellent review on vagus nerve physiology see* Yuan and Silberstein, 2016a,b). Three types of fibers have been identified, each with distinct physiological properties. In general, the larger the fiber, the faster the conduction speed. Myelinated A-fibers are composed of small and large fibers. The small fibers are visceral afferent fibers and the large are both afferent and efferent somatic fibers. Afferent and efferent preganglionic fibers are called B-fibers. Finally, ~70% of all vagal fibers are unmyelinated C-fibers and convey visceral information from the vast array of visceral organs. Acetylcholine is the primary neurotransmitter of the vagus nerve. It activates cholinergic receptors that are subdivided into nicotinic and muscarinic receptors. However, there is evidence of cross-talk between the vagus and sympathetic nerve fibers as evidenced by tyrosine hydroxylase in the thoracic and cervical trunks of the vagus. There are four vagal nuclei in the medulla, each with distinct but often overlapping targets. The nucleus ambiguus is the source of most cardiovagal motor neurons. The dorsal motor nucleus also contains some cardiovagal motor neurons but primarily innervates the subdiaphragmatic visceral organs. The nucleus of the solitary tract (NTS) is the major hub for afferent information. Finally, the spinal nucleus of the trigeminal nerve, via the superior jugular ganglion, transmits afferent and efferent impulses primarily from the head and vocal structures and has several branches including the auditory branch (Yuan and Silberstein, 2016a). Furthermore, the vagus nerve has projections to higher brain centers including the prefrontal cortex primarily via synaptic connections in the NTS (Thayer and Lane, 2009). In addition, there may be variation among species in the anatomy and physiology of the vagus requiring comparisons of studies across species to be done mindfully. An understanding of the complex anatomy and physiology of the vagus nerve is essential to an understanding of vagus nerve stimulation.

According to the reports of historians and archaeologists, clinical applications of auricular stimulation (broadly defined) were used across many ancient cultures. For instance, tactile stimulation of the auditory meatus was mentioned in some of the earliest known texts on Chinese medicine and acupuncture (Hou et al., 2015). Interestingly, therapeutic auricular stimulation was not confined to China, and was prevalent across many cultures. Thousands of years ago, the practice of cauterizing a portion of the auricle was common amongst certain tribes in Arabia, while in ancient Egypt, women pricked or cauterized the external auricle for contraceptive purposes and physicians in ancient Persia treated sciatic pain and sexually-related diseases by auricular cauterization (Hou et al., 2015). The Italian anatomist and surgeon Antonio Valsalva published his famous Tractatus de Aure Humana, where he described the treatment of toothache by scarification of the antitragus (Valsalva, 1704). In the last half of the twentieth century, the auricular acupuncture (i.e., needling of specific areas of external auricle) became popular in clinical medicine (Nogier, 1957). Based on Nogier's work in the German Journal of Acupuncture 1957, the Nanjing Army Ear Acupuncture Research Group from China further evaluated auricular somatotopy (Huang, 1974), and auricular acupuncture developed as a unique “microsystem” for acupuncture therapy. Currently, auricular acupuncture, which can mimic transcutaneous vagus nerve stimulation (tVNS), is reported in numerous systematic reviews to be effective in treatment of insomnia and relief of acute and chronic pain (Vieira et al., 2018). Ultimately, there is a great deal of overlap between acupuncture, particularly electroacupuncture, and neuromodulation therapies such as tVNS (Usichenko et al., 2017b), and the rich evidence base supporting auricular acupuncture should be better integrated to help inform further development of tVNS therapy (Napadow, 2019).

The origins of VN stimulation (VNS) date back in excess of 100 years. In the late eighteenth and early nineteenth century, it was believed that epilepsy was caused by excessive blood flow to the brain, termed venous hyperaemia, with patients frequently being treated by manual compression of the carotid arteries in the neck to suppress blood flow. In the late nineteenth century, American neurologist *James L. Corning* developed a “carotid fork”—a device to facilitate carotid compression, which was later augmented by stimulation electrodes. Corning intended to stimulate cervical branches of the VN, which course in close proximity to the carotid artery, in order to decrease heart rate (HR) and, subsequently, blood flow to the brain. Even though Corning reported treatment success, the method was not widely accepted at the time due to safety concerns and a lack of reproducibility of therapeutic response (Lanska, 2002).

Implantable VNS (iVNS) was developed by Jake Zabara in the 1980s as it was found to have promising antiepileptic effects in canine models (Zabara, 1985, 1992) and proceeded to become one of the earliest forms of neuromodulation in humans (Yuan and Silberstein, 2016a). Globally, by 2014 over 100,000 patients have had iVNS implanted (Johnson and Wilson, 2018; *also see* Chakravarthy et al., 2015). The first controlled clinical trials of iVNS as a treatment for refractory epilepsy were conducted in the early 1990s (Penry and Dean, 1990; Uthman et al., 1993) and reported substantial reductions in seizure frequency, even though a significant proportion of patients did not display a symptomatic improvement. Following a number of further clinical trials, iVNS, applied to the left cervical VN, was approved by the US Food and Drug Administration (FDA) for management of pharmacoresistant epilepsy in 1997 (Morris et al., 2013). In subsequent observational studies of patients with epilepsy, it was reported that patients' mood improved following iVNS treatment (Harden et al., 2000). These results spawned a series of studies in patients with depression which led, in 2005, to FDA approval of iVNS for the treatment of pharmacoresistant depression (Cristancho et al., 2011; Desbeaumes Jodoin et al., 2018). More recently, iVNS has been evaluated as treatment for a diverse array of disorders including heart failure (Ferrari et al., 2011; Wang et al., 2015a), rheumatoid arthritis (Koopman et al., 2016), inflammatory bowel disease (Levine et al., 2014), sepsis (Wang et al., 2016; Yang et al., 2017), and chronic pain (Lange et al., 2011).

iVNS necessitates a costly, invasive surgical procedure involving the implantation of a bipolar helical electrode to the left cervical VN which is subsequently attached to a pulse generator, most frequently positioned in a left infraclavicular subcutaneous pocket. Non-invasive tVNS approaches have been developed as a less expensive, patient friendly and rapidly deployable alternative. Transcutaneous cervical VNS (tcVNS) is conceptually similar to Corning's initial approach of transcutaneously stimulating the VN in the neck, adjacent to the carotid artery. tcVNS is FDA-approved for the treatment of migraine and cluster headache management and has been subjected to intensive research effort (Goadsby et al., 2014; Nesbitt et al., 2015).

**Figure 1:**
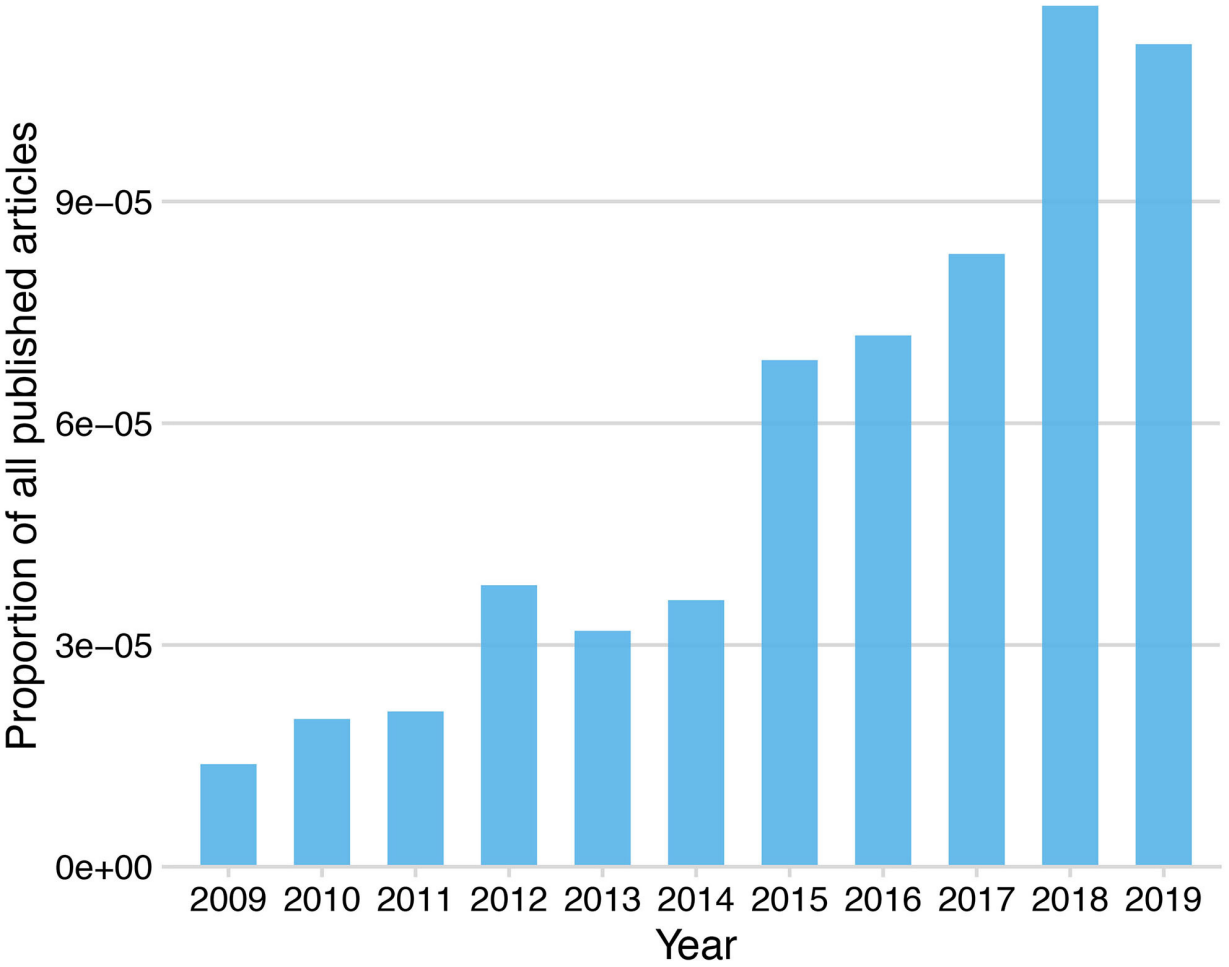
Proportion of published research articles including the keyword “transcutaneous vagus nerve stimulation” listed on PubMed by year.

The most widely commercially available tcVNS device (gammaCore®, electroCore, Inc.) is hand-held and delivers sinusoidal alternating current with a broadband amplitude-modulated frequency spectrum (Nesbitt et al., 2015). In the USA, the gammaCore® device received FDA approval for the treatment of acute cluster headache treatment in 2017, and for acute migraine treatment and adjunctive cluster headache prevention in 2018. Transcutaneous auricular VNS (taVNS) is under investigation for a wide range of clinical applications, however, is not FDA-cleared for the treatment of any disorder. The most widely used commercially available taVNS device (NEMOS®, tVNS technologies) delivers current in rhythmic square pulses (Yuan and Silberstein, 2016b). The NEMOS® device received European certification (CE certification, which indicates legal conformity and safety, but not necessarily clinical efficacy) as a treatment for epilepsy and depression in 2010, for chronic pain in 2012 and for anxiety in 2019. Importantly, given their non-invasiveness, tcVNS and taVNS are widely used not only for clinical purposes, but also in healthy populations for basic research in cognitive neuroscience and related fields (Yuan and Silberstein, 2016b). The increased availability of these devices, coupled with their user friendliness, has resulted in an increase in research publications on tVNS, see Figure 1.

An array of stimulation parameters needs to be considered when it comes to using tVNS in both research and clinical settings. Stimulation parameters of tVNS can vary in terms of its current intensity (mA), pulse width (μs), frequency (Hz), duty cycle (s), and session duration (min) (Badran et al., 2019). Furthermore, side effects of stimulation, type of sham or control stimulation, location of the stimulation and sham electrode placement may influence the outcomes of tVNS. The impact of each of these stimulation parameters on psychophysiology and on clinical outcomes is incompletely understood. Despite the increasing number of studies, there is no clear consensus regarding the optimal parameters that need to be adopted for tVNS research. Moreover, there is no clear consensus regarding the minimal standard reporting items within the tVNS literature. Recently, calls for full disclosure of tVNS stimulation parameters have been made (Redgrave et al., 2018; Burger et al., 2020a). Herein, we aim to provide multidisciplinary recommendations regarding standard reporting items for future tVNS research. These recommendations are based on a systematic review of existing tVNS studies, evaluation of current reporting practices and finally on a broad consensus among research groups studying tVNS.

### VNS Nomenclature: Techniques and Targets

The following section reviews four currently accepted VNS modalities—(1) cervically implanted VNS (iVNS), (2) transcutaneous cervical VNS (tcVNS), (3) transcutaneous auricular VNS (taVNS) (4) percutaneous auricular VNS (paVNS). For simplicity, hereinafter, we will refer to both transcutaneous forms of VNS (taVNS and tcVNS) as tVNS.

#### Cervically Implanted VNS (iVNS)

Classically, the VN is stimulated via implanted electrodes targeting (mostly) the left cervical branch of the VN (Mertens et al., 2018; Kaniusas et al., 2019a). iVNS commonly uses a bipolar cuff electrode (e.g., VNS Therapy, LivaNova). Despite being well-established, this method remains expensive and is associated with peri and post implantation risks. Furthermore, the electrode implant is irreversible. Moreover, stimulation is not restricted to afferent fibers of the cervical VN—as usually targeted by the therapy—but extends to (visceral) efferent fibers of the VN as well (Howland, 2014). Consequently, several adverse effects such as cough, voice alteration, swallowing difficulties, or bradycardia have been reported (Liporace et al., 2001).

#### Transcutaneous Cervical VNS (tcVNS)

The cervical VN can also be stimulated transcutaneously by using two skin electrodes, e.g., by a hand-held device (e.g., GammaCore, electroCore, Inc.), which are applied at the neck (Barbanti et al., 2015; Gaul et al., 2016; Silberstein et al., 2016a; Frangos and Komisaruk, 2017). This form of transcutaneous stimulation is now FDA approved for the acute treatment of migraine and for the acute treatment and prevention of episodic cluster headache. However, despite its relative convenience, this method is not devoid of adverse effects. Given that tVNS requires the stimulation to pass through the skin barrier, relatively strong currents are needed. The resulting stimulation fields in the neck are diffuse, so that cervical non-vagal nerves can be co-stimulated, as well as efferent cervical fibers. Commonly observed adverse effects for the cervical tVNS approach include prickling at the stimulation site, neck pain, dizziness, headache, nasopharyngitis, oropharyngeal pain and sensitivity to the conducting gel (Gaul et al., 2016; Redgrave et al., 2018). An MRI-derived *Finite Element Method* model was developed to analyze the cellular components activated with tcVNS. Due to the different types of tissue between the surface electrodes on the skin and the VN, both macroscopic (skin, muscle, fat) and mesoscopic (nerve sheath, cerebrospinal fluid) components were used to predict activation thresholds and electric field changes. It was demonstrated that the overall current requirement to achieve adequate stimulation is influenced by deeper tissues and that tissue conductivity has a direct effect on axon membrane polarization. This model predicts that tcVNS will activate A and B axon fibers, but not C fibers (Mourdoukoutas et al., 2018).

#### Transcutaneous Auricular VNS (taVNS)

The auricular branch of the VN is primarily an afferent fiber which innervates the ear and joins the main bundle of the VN projecting to the nucleus tractus solitarius (NTS). taVNS is achieved via surface skin electrodes applied in the vagally-innervated ear regions (Ellrich, 2011) on the outer ear (Ellrich, 2011; Frangos et al., 2015; Straube et al., 2015; Badran et al., 2018a,b). Typically, taVNS uses two surface electrodes (e.g., NEMOS, tVNS Technologies GmbH). This method is CE marked (but not FDA approved) for epilepsy, depression, anxiety, pain, and migraine. A relatively large surface of electrodes yields diffuse stimulation fields, so that not only vagal but also non-vagal auricular nerves can be recruited, the implications of which remain controversial (Kaniusas et al., 2019a). The stimulation is considered safe (Badran et al., 2018c). As in the case of the percutaneous tcVNS, the expected side effects are mostly minor and may include headache, pain and skin irritation at the stimulation site, and dizziness (Mertens et al., 2018). Researchers are still working to determine optimal ear targeting approaches as there is paucity of data comprehensively describing the innervation of the ear. An anatomical dissection of the human auricle describes how the auricular branch of the VN diffusely innervates the ear (Peuker and Filler, 2002), with the cymba concha region being exclusively innervated by the auricular branch of the VN, along with other areas such as the posterior and inferior walls of the ear canal. Many of these targets are hypothesized to be regions for engagement of vagal afferents (Badran et al., 2018a; Burger and Verkuil, 2018). Various ear targets, practical procedures and electrode placement techniques for taVNS in the laboratory or clinical setting have been outlined along with stimulation parameter considerations (Badran et al., 2019; Sclocco et al., 2019, 2020).

#### Percutaneous Auricular VNS (paVNS)

A minimally invasive form of paVNS (Kampusch et al., 2013) can be performed with miniature needle electrodes penetrating the skin in the targeted outer ear regions innervated mainly by the auricular branch of the VN (Sator-Katzenschlager et al., 2004; Kaniusas et al., 2019b). This so-called paVNS typically uses 2–3 needle electrodes (e.g., AuriStim, DyAnsys). The small size of needle electrodes and the resulting spatially focused stimulating fields favors precise and specific stimulation of the local afferent auricular branch VN endings. Minor side effects of paVNS are local skin irritation (dermatitis), local bleeding, pain at the stimulation site, and dizziness.

Thus, the term tVNS is a broadly encompassing term and is not location-specific, i.e., neck or ear, as both tcVNS and taVNS may have similar biological effects. It is important to note there is limited data on the head-to-head testing of tcVNS with taVNS and these studies should be explored.

In the following sections, we will focus on tVNS, given that these techniques have been subject to intensive study. The key difference between taVNS and tcVNS is the branch of the VN which is putatively targeted. taVNS targets the auricular branch of the VN, a subsidiary from both left and right VN main bundles that innervates the ear on the same side (Peuker and Filler, 2002). In contrast, tcVNS targets the cervical branch of the VN (Barbanti et al., 2015; Gaul et al., 2016; Silberstein et al., 2016b; Frangos and Komisaruk, 2017). It remains unclear whether neck and ear stimulation produce similar biological and end organ effects.

tVNS can also be associated with indirect sporadic effects, due to rare afferent-efferent vagal reflexes via the NTS. For instance, tVNS may sometimes cause a reflexive cough, colloquially known as Arnold's ear-cough reflex. Other vegetative reflexes, such as the ear-gag reflex, ear-lacrimation reflex, ear-syncope reflex, and vaso-vagal reflex can also be observed, albeit relatively rarely (Tekdemir et al., 1998; Ellrich, 2011; Napadow et al., 2012). Overall, tVNS is associated with fewer side effects in comparison to iVNS, which has the potential to be associated with increased tolerability. In addition, portable devices are relatively easy to handle and are more cost effective than implantable devices (Morris et al., 2016).

## Modes of Application

### Long-Term Stimulation in Clinical Trials and Intervention Studies

#### Epilepsy

The effect of tVNS on pharmacoresistant epilepsy has been investigated in several studies. An early pilot study (Stefan et al., 2012) demonstrated that seizure frequency was reduced in five out of seven patients after 9 months of tVNS therapy, and that tVNS was well-tolerated. This reduction was also observable in a larger sample size over a 12 months period (Aihua et al., 2014). Another 6 months pilot study (He et al., 2013a) demonstrated seizure frequency reductions in 9 out of 14 children. A more recent 20-week placebo-controlled clinical trial of 76 patients with epilepsy (Bauer et al., 2016) reported that tVNS decreased seizure frequency. However, only about half of the patients were classified as responders—defined as seizure frequency reduction >25%. In a more recent prospective study of 20 patients (Barbella et al., 2018), only one third derived clinical benefit from tVNS. In a randomized clinical trial of 47 patients with epilepsy, Rong et al. reported that after 24 weeks of daily treatment 16% were seizure free and 38% had reduced seizure frequency (Rong et al., 2014). A larger-scale clinical trial of tVNS in epilepsy is pending, as the evidence regarding efficacy is currently insufficient for routine clinical care (Boon et al., 2018). Although the mechanisms of VNS in epilepsy are not fully understood, it is suggested that the nuclei of the brainstem are involved. The NTS has direct or indirect projections toward the locus coeruleus (LC) and raphe nuclei, which are suggested to be associated with seizures through noradrenergic and serotonergic neurons, exerting an antiepileptic effect. In particular, antiepileptic effects have been associated with an increase in norepinephrine (Krahl and Clark, 2012; Panebianco et al., 2016). Another theory suggests that VNS can activate inhibitory structures in the brain, with an increase in free gamma-aminobutyric acid (GABA) levels in cerebrospinal fluid and GABA-A receptor density in the hippocampus of patients who responded favorably (Marrosu et al., 2003). In recent years, the idea has grown that inflammation is involved in the development of seizures and epilepsy and, therefore, activation of anti-inflammatory pathways through VNS could explain antiepileptic effects (Krahl and Clark, 2012; Bonaz et al., 2013; Panebianco et al., 2016). Early work in rats indicated that the recruitment of vagal C-fibers is necessary for the suppression of seizures, by activating the C-fibers of the Vagal nerve (Woodbury and Woodbury, 1990), mediating GABA and glycine levels.

#### Depression

A placebo-controlled pilot study of patients with depression (Hein et al., 2013) found that 2 weeks of tVNS decreased depression severity using validated measures. This finding was replicated later in a larger patient sample, although this non-randomized study identified only about one third of the patients enrolled as tVNS responders (Rong et al., 2016). Neuroimaging studies in mild to moderately depressed patients have demonstrated that tVNS altered functional brain connectivity in the default mode network (Fang et al., 2016; Liu et al., 2016) and led to insula activation that was correlated with the clinical effectiveness of tVNS treatment (Fang et al., 2017). Furthermore, a decrease in functional connectivity between the bilateral medial hypothalamus and rostral anterior cingulate cortex (rACC) (Tu et al., 2018), as well as an increase of functional connectivity between the left nucleus accumbens and bilateral rACC (Wang et al., 2018) during 4 weeks of tVNS treatment were reported. Another potential mechanism, by which tVNS may exert an antidepressant action, is the modulation of inflammatory processes that are currently discussed (Rawat et al., 2019; *also see* Pavlov and Tracey, 2012). A previous review has summarized existing research on tVNS in depression in greater detail (Kong et al., 2018 also see Lv et al., 2019).

#### Tinnitus

A third clinical field in which several tVNS studies exist is tinnitus. One pilot study (Lehtimäki et al., 2013) found that 10 days of tVNS, combined with sound therapy, ameliorated patient-reported tinnitus severity and attenuated their auditory event-related field signal on magnetoencephalography (MEG). Another pilot study similarly observed a clinically meaningful amelioration of patient-reported tinnitus severity in four out of 10 patients after 20 days of combined tVNS and sound therapy (De Ridder et al., 2014). This has been replicated in a larger sample (30 patients), 15 of which were classified as responders to combined tVNS and sound therapy (Shim et al., 2015). However, a further pilot study administering tVNS (without sound therapy) for 6 months did not show any clinically meaningful effect (Kreuzer et al., 2014). It appears that the VNS generates improvements in patients with tinnitus due to the suppression of auditory, limbic and other areas of the brain involved in the generation / perception of tinnitus through the ascending auditory and vagal pathways (Yakunina et al., 2018). The rationale for the treatment of tinnitus using tVNS is build around the idea, that tVNS together with the presentation of tones can boost neuronal plasticity: the joint use of VNS and tones produces a reduction in the activity of the gamma band in the left auditory cortex, as well as the phase coherence between the cortex. Auditory and areas associated with tinnitus distress, including the cingulate cortex (Vanneste et al., 2017).

#### Other Clinical Conditions

The effect of tVNS on a variety of other diseases has been explored. A pilot study of tVNS in schizophrenia found no effect on symptom severity (Hasan et al., 2015). Moreover, many potential targets for treatment via tVNS have been suggested, including attention deficit hyperactivity disorder (ADHD) (Beste et al., 2016), autism spectrum disorders (Jin and Kong, 2016), Alzheimer's dementia (Jacobs et al., 2015), post-operative cognitive dysfunction (Xiong et al., 2009), increased risk of type II diabetes (Huang et al., 2014), preterm infants with oromotor dysfunction (Badran et al., 2020), chronic stroke patients (Capone et al., 2017), coronary insufficiency (Afanasiev et al., 2016) and chronic migraine (Straube et al., 2015). The idea that tVNS might be a promising treatment in Alzheimer's dementia has received support through recent evidence that tVNS can recover impaired microglia function in a mouse model of Alzheimer's dementia (Kaczmarczyk et al., 2017; Huffman et al., 2019), and there is an ongoing clinical trial of tVNS as a treatment for mild cognitive impairment (NCT03359902). For ADHD, trigeminal nerve stimulation (TNS) has been suggested as a complementary treatment to tVNS, and a recent study found promising clinical improvements (McGough et al., 2019). A study in patients with chronic pelvic pain (Napadow et al., 2012) found that tVNS ameliorated patient-reported pain intensity and anxiety. Antinociceptive effects of tVNS have been replicated in some studies but not in others, and its effect has remained inconsistent between studies and individuals (Laqua et al., 2014; Usichenko et al., 2017a,b; De Icco et al., 2018; Janner et al., 2018).

Whilst the above studies assume that the effects of tVNS are primarily mediated by central neuromodulation, i.e., effects on neurotransmission and neuroplasticity in the brain, tVNS-induced cardiovagal and cardiosympathetic effects have also been reported, and a number of studies have focused on the clinical potential of these effects. For example, a number of studies have found tVNS to reduce sympathetic nerve activity, indexed through resting muscle sympathetic activity (Clancy et al., 2014; Murray et al., 2016b; Ylikoski et al., 2017). However, cardiac effects of tVNS may be related to stimulation parameters, such as pulse width and stimulation frequency (Badran et al., 2018c) and there remains to date unexplained inter-individual variations in the clinical response to these parameters in the treatment of cardiovascular disease (Murray et al., 2016a).

Taken together, these studies indicate that tVNS has the potential to treat a wide range of clinical conditions. One of the key challenges for its further development appears to be the lack of inter-individual consistency in treatment success. Those differences are currently not well-understood, and may relate to anatomical differences, physiological state, and stimulation parameters. Table 1 provides an overview of the stimulation parameters that have been deployed in various studies along with other characteristics of those reports^1^.

**Table 1:** Reported stimulation parameters in studies on long-term tVNS.

**References**	**Clinical entity**	**N/** **clinical trial**	**Device**	**Electrode(s)**	**Electrode placement**	**Stimulation length**	**Alternating stimulation**	**Stimulus intensity**	**Pulse width**	**Hz**	**Results**
Hein et al. (2013)	Depression	37 MDD patients **Study 1**: 22 (11 sham vs. 11 auricular) **Study 2**: 15 (6 once vs. 9 twice a day of stimulation)	**Study 1**: TENS microstimulator unit NET-2000 made by Auri-Stim Medical, Inc., 11172 Huron St. Suite 22, Denver, CO, USA **Study 2**: NET-1000 (self-application by the patients) also made by Auri-Stim Medical		**Study 1**: On both sides, four electrodes were placed crosswise, each with a diameter of about 3 mm Sham: no current **Study 2**: on both sides Sham: manipulated clamp, no current	**Study 1**: 15 min once for 2 weeks on 5 days each week **Study 2**: 15 min twice a day for 2 weeks on 5 days each week		**Study 1**: 0–max. 600 μA **Study 2**: 130 μA		**Study 1**: 1.5 Hz unipolar rectangle waves **Study 2**: 1.5 Hz	2 weeks tVNS resulted in decreased depression severity
Fang et al. (2016) Fang et al. (2017)	Depression	49 MDD patients single-blinded clinical trial	Ear vagus nerve stimulator Institute of Acupuncture and Moxibustion, China Academy to Chinese Medicine Science (Beijing, China) and Suzhou Medical Appliance Factory (Jiangsu Province, China)	Special ear clips (electrodes) (Huang et al., 2014; Rong et al., 2014)	tVNS Auricular conchae Sham Superior scapha	30 min, twice a day, at least 5 days per week for 4 weeks		4–6 mA	2016: <1 ms 2017: 0.2 ms	20 Hz continuous sinusoidal wave	4 weeks tVNS resulted in decreased depression severity tVNS modulates DMN FC
Liu et al. (2016)	Depression	49 MDD patients single-blinded clinical trial	[…] full details of the study are reported elsewhere (Fang et al., 2016; Rong et al., 2016)	[…] full details of the study are reported elsewhere (Fang et al., 2016; Rong et al., 2016)	Applied on both ears simultaneously during treatment tVNS Auricular conchae Sham Superior scapha	30 min, twice a day (morning, evening), at least 5 days per week for 4 weeks **First cohort:** 12 weeks tVNS **Second cohort:** 4 weeks sham and 8 weeks real tVNS		4–6 mA	<1 ms	20 Hz	4 weeks tVNS resulted in decreased depression severity tVNS modulates amygdala-lateral prefrontal rsFC
Rong et al. (2016)	Depression	MDD patients non-randomized, controlled study **First cohort:** *N* = 91 **Second cohort:** *N* = 69	Ear vagus nerve stimulator Institute of Acupuncture and Moxibustion, China Academy to Chinese Medicine Science (Beijing, China) and Suzhou Medical Appliance Factory (Jiangsu Province, China)	Special ear clips (electrodes) (Huang et al., 2014; Rong et al., 2014)	tVNS Auricular conchae Sham Superior scapha	**First cohort:** 12 weeks tVNS **Second cohort:** 4 weeks sham tVNS and 8 weeks real tVNS		4–6 mA	0.2 ms	20 Hz continuous sinusoidal wave	Greater symptom reductions during tVNS for the first 4 weeks
Tu et al. (2018)	Depression	41 MDD patients Non-RCT, single-blinded clinical trial	See Ack “[…] supported by […] Chinese Medicine […] Beijing Natural Science […]”	See Ack “[…] supported by […] Chinese Medicine […] Beijing Natural Science […]”	tVNS Auricular conchae Sham Superior scapha	30 min, twice a day (morning, evening) at least 5 days per week, for 4 weeks		4–6 mA	<1 ms	20 Hz	4 weeks tVNS resulted in decreased depression severity During tVNS decreased FC between MH and rACC
Wang et al. (2018)	Depression	41 MDD patients single-blinded, non-randomized clinical study	Ear vagus nerve stimulator Institute of Acupuncture and Moxibustion, China Academy to Chinese Medicine Science (Beijing, China) and Suzhou Medical Appliance Factory (Jiangsu Province, China)	Special ear clips (electrodes) (Huang et al., 2014; Rong et al., 2014)	tVNS Both ears during treatment (during MRI right ear) Suricular conchae Sham Superior scapha	**First cohort:** 12 weeks tVNS **Second cohort:** 4 weeks sham tVNS and 8 weeks real tVNS		4–6 mA	<1 ms	20 Hz continuous sinusoidal wave	During tVNS increased FC between left NAc and MPFC/rACC and negative correlation with changes in symptom severity
Aihua et al. (2014)	Epilepsy	60 pharmacoresistant epilepsy patients (50% sham) Randomized controlled trial	TENS-200, Hua Tuo brand		Bilateral tVNS (*N* = 26) Ramsay-Hunt Zone Sham (*N* = 21) Earlobe	Three times per day, continuous stimulation for 20 min, for 12 months		Median (IQR) 6 mA	0.2 s	30 Hz	Reduced seizure frequency after 12 months of daily tVNS
Barbella et al. (2018)	Epilepsy	20 patients with refractory focal epilepsy, drug resistant	NEMOS (cerbomed GmbH, Erlangen, Germany) not cited because the only device used at the time in Italy for tVNS		Left auricolar concha, not cited, but considered by défault the site of electrodes!	4 h per day, divided into two-three sessions of at least 1 h each, for 6 months	20 s on/ 5 min off	0.6–0.8 mA			Reduced seizure frequency in about one third of the patients after 6 months of daily tVNS
Bauer et al. (2016) Hamer et al. (2019)	Epilepsy	76 drug-resistant epilepsy, randomized, double-blind clinical trial Low level: 1 Hz *N* = 39 High level: 25 Hz *N* = 37	NEMOS (cerbomed GmbH, Erlangen, Germany) CE certified tVNS device		Left auricular branch of the vagus nerve at the ear conch	4 h daily, for a period of 20 weeks +8-weeks baseline period	30 s on/30 s off	High level: 0.50–0.47 mA Low level: 1.02–0.83 mA	High level: 250 us Low level: 250 us	High level: 25 Hz Low level: 1 Hz	Reduced seizure frequency after 20 weeks of daily tVNS
He et al. (2013a)	Epilepsy	14 pediatric patients with intractable epilepsy	TENS-200, Suzhou, China	Two pairs of electrode clips, made of conductive rubber, 5 mm in diameter	ta-VNS 1 × concha cavity 1 × concha cymba	Three times a day, 30 min per session, 24 weeks		0.4–1.0 mA		20 Hz	Reduced seizure frequency in nine out of 14 patients during 6 months of tVNS therapy
Rong et al. (2014)	Epilepsy	50 patients with drug-resistant epilepsy, random clinical trial	TENS, Suzhou Medical Appliance Co. Ltd., Suzhou, China	Electrode clamp with two carbon-impregnated silicone electrode tips connected to the TENS by metal wires for electrical stimulations		Twice times a day, 30 min per session, 24 weeks		1 mA	<1 ms	20–30 Hz	Reduced seizure frequency and seizure free patients after 6 months
Stefan et al. (2012)	Epilepsy	10 patients with pharmacoresistant epilepsy, pilot study		Stimulus area ~2 cm^2^	tVNS Left ear	Three times per day (1 h in morning, noon, evening), for 9 months		Mean 25 V	300 μs	10 Hz biphasic	Reduced seizure frequency in five out of seven patients after 9 months of tVNS therapy
Bretherton et al. (2019) Study 3	HRV	26 older participants	TENS machine (EMS7500 Roscoe Medical)	Customized auricular electrode clips	Inner and outer surface of the tragus of the ear (Auricular Clips, Body Clock Health Care Ltd, UK)	15 min once daily for 2 weeks		2–4 mA	200 μs	30 Hz	Improvement of autonomic function, health-related QoL, mood, sleep after 2 weeks of daily tVNS
Huang et al. (2014)	Impaired glucose tolerance	72 participants with IGT pilot randomized clinical trial	TENS-200 (developed by Suzhou manufacture of Medical Device and Material)		tVNS Auricular conchae Sham Superior scapha	Twice a day, post-prandial treatment lasted 20 min, half an hour after eating, for 12 weeks		1 mA	≤1 Hz	20 Hz	Reduced systolic blood pressure after 12 weeks of daily tVNS
Straube et al. (2015)	Migraine	46 chronic migraine patients Monocentric, randomized, controlled, double-blind study **1 Hz** *N* = 22 **25 Hz** *N* = 24	NEMOS® taVNS device (Cerbomed, Erlangen, Germany)		Concha of outer ear	4 h per day (free to stimulate for additional hour) for 12 weeks	30 s on 30 s off	Individually fitted, adjustment by patient if it was needed	250 μs	1 Hz 25 Hz	1 Hz group had a significantly larger reduction in headache days per 28 days than patients in the 25 Hz group
Hasan et al. (2015)	Schizophrenia	25 schizophrenia patients, bicentric randomized, sham-controlled, double blind pilot study **Group 1**: active tVNS **Group 2**: sham tVNS	CM02, Cerbomed, Erlangen, Germany	Two titan electrodes	Left auricle branch of vagus nerve	Daily stimulation for 26 weeks 1. settling-in phase (3 × 1 h/day) 2. adaption phase 1 (3 × 2 h/day) 3. adaption phase 2 (3 × 3 h/day) Advised to use stimulator whole day **Group 1**: 12 weeks active 14 weeks sham	30 s on 180 s off Duty cycle 14%	0.1–10 mA	250 μs	25 Hz	No improvement of schizophrenia symptoms in 26-weeks tVNS trial
						**Group 2**: 12 weeks sham 14 weeks active					
Capone et al. (2017)	Stroke	14 patients with ischemic or hemorrhagic chronic stroke, randomized (tVNS vs. sham)	Electric stimulator (Twister—EBM)	2 Ag-AgCl electrodes (5 mm in diameter)	**tVNS** left external acoustic meatus at the inner side of the tragus **Sham** earlobe(left)	Stimulation repeated every 5 min for 60 min, for 10 days	30 s	**tVNS** Mean = 2.0–4.5 **Sham** Mean = 2.8–7.2	0.3 ms	20 Hz	tVNS and robotic rehabilitation can improve arm functionality in chronic stroke patients
Kreuzer et al. (2014)	Tinnitus	50 patients with chronic tinnitus, open single-armed pilot study **Phase 1** *N* = 24 **Phase 2** *N* = 26 (new)	**Phase 1** Cerbomed CM02 (Erlangen, Germany) **Phase 2** NEMOS (Erlangen, Germany)			24 weeks **Phase 1** For at least 6 h per day **Phase 2** 4 h per day	**Phase 1** 30 s on 180 s off **Phase 2** 30 s on 30 s off	0.1–10 mA		25 Hz	No clinically meaningful effect after 6 months pf tVNS
Lähtimäki et al. (2013)	Tinnitus	10 patients with tinnitus, pilot study, short-term tVNS and sound therapy	Tinnoff pulse generator (Jarmo Lehtimäki is an employee and Matti Ylikoski and Jukka Ylikoski are board members of Tinnoff Inc.)	Clip electrode	Auricular branch of vagus nerve, clip at left tragus	Seven sessions, each 45–60 min, for 10 days		>0.8 mA		25 Hz	10 days of tVNS ameliorated patient-reported tinnitus severity
Shim et al. (2015)	Tinnitus	30 patients with refractory chronic tinnitus	TENS eco2 (Schwa-medico, Ehringshausen, Germany)	Silicon electrical pad (2 cm in diameter)	Auricular concha of the patient's left ear	10 sessions intervals of 1–4 days		1–10 mA	200 μs	25 Hz	50% reported symptom relief after 10 tVNS sessions

### Acute/Short-Term Stimulation in Experimental Trials

Alongside clinical trials and intervention studies, tVNS has gained increasing interest as a tool for neuromodulation in experimental studies. Based on evidence that vagal activity is related to a host of psychological and physiological processes, tVNS promises deeper insights by enabling active manipulation of VN activity. Predominantly, these studies are characterized by short stimulation periods, addressing the immediate effects. Psychological targets have been broad (though not all of them are sensitive to tVNS), including: experimentally induced worry (Burger et al., 2019a); post-error slowing (Sellaro et al., 2015b); attention to fearful faces (Verkuil and Burger, 2019); associative memory (Jacobs et al., 2015) or single-item word memory (Giraudier et al., 2020; Mertens et al., 2020); extinction of fear responses or fear conditioning (Burger et al., 2016, 2017, 2018, 2019b; Genheimer et al., 2017; Szeska et al., 2020); implicit spiritual self-representations (Finisguerra et al., 2019); flow experience (Colzato et al., 2018b); response selection during sequential action (Jongkees et al., 2018) or during action cascading processes (Steenbergen et al., 2015); the recognition of emotions in faces or bodies (Colzato et al., 2017; Sellaro et al., 2018; Koenig et al., 2019); divergent thinking (Colzato et al., 2018a); conflict-triggered adjustment of cognitive control (Fischer et al., 2018); auditory selective attention (Rufener et al., 2018) or visual selective attention (Ventura-Bort et al., 2018); inhibitory control (Beste et al., 2016; Borges et al., 2020); automatic motor inhibition (Keute et al., 2018); cognitive flexibility (Borges et al., 2020; Tona et al., 2020); prosocial behavior (Sellaro et al., 2015a) and reward sensitivity (Neuser et al., 2019).

Other more physiologically oriented studies have investigated the influence of tVNS on cardiac activity (Brock et al., 2017; De Couck et al., 2017; Lamb et al., 2017; Gancheva et al., 2018; Borges et al., 2019; Bretherton et al., 2019; Koenig et al., 2019; Paleczny et al., 2019; Tobaldini et al., 2019; Tran et al., 2019); autonomic outflow (Sclocco et al., 2017); sympathetic nerve activity (Clancy et al., 2014) or cardiac baroreflex sensitivity (Antonino et al., 2017); atrial fibrillation (Stavrakis et al., 2015); cardiac mechanical function (Tran et al., 2019); vagal sensory evoked potentials (Fallgatter et al., 2003, 2005; Polak et al., 2009; Leutzow et al., 2013); persistent hiccups (Schulz-Stübner and Kehl, 2011); visual bistable perception (Keute et al., 2019a); nociceptive neuromodulation (Napadow et al., 2012; Busch et al., 2013; Laqua et al., 2014; Usichenko et al., 2017b; Janner et al., 2018); tumor necrosis factor-alpha (Brock et al., 2017); hepatic energy metabolism (Gancheva et al., 2018); whole blood culture-derived cytokines and chemokines (Lerman et al., 2016); salivary hormones (Ventura-Bort et al., 2018; Koenig et al., 2019; Warren et al., 2019); pupil diameter (Warren et al., 2019); gastroduodenal or gastrointestinal motility (Frøkjaer et al., 2016; Juel et al., 2017); muscle activity in the gastrointestinal tract (Hong et al., 2019), gastric frequency (Teckentrup et al., 2020); electroencephalography (Hyvärinen et al., 2015; Keute et al., 2018; Lewine et al., 2019) and event-related potentials (Lewine et al., 2019), specifically the P3/P300 event-related potential (Ventura-Bort et al., 2018; Warren et al., 2019); cortical excitability (Capone et al., 2015) and changes in blood-oxygen-level-dependent (BOLD) functional Magnetic Resonance Imaging (fMRI) (Kraus et al., 2007, 2013; Dietrich et al., 2008; Frangos et al., 2015; Frangos and Komisaruk, 2017; Garcia et al., 2017; Yakunina et al., 2017, 2018; Badran et al., 2018b; Peng et al., 2018; Sclocco et al., 2019, 2020). Ultrahigh field (7T) fMRI studies with enhanced spatiotemporal resolution have clearly demonstrated tVNS stimulus-evoked activation of the ipsilateral NTS, the primary synapse for vagus nerve traffic to the brain (Garcia et al., 2017; Sclocco et al., 2019, 2020). Cases reports illustrate the use of tVNS in the treatment of a patient with persistent geotropic direction-changing positional nystagmus (Cha et al., 2016) or insomnia (Yu et al., 2017). Table 2 summarizes the characteristics of these studies on acute/short-term tVNS.

**Table 2:** Reported stimulation parameters in studies on acute/short-term tVNS.

**References**	**Device**	**Electrode(s)**	**Stimulus intensity**	**Pulse width**	**Hz**	**Alternating stimulation**	**Stimulus**	**Electrode placement**	**Stimulation length**	**Task**	**Pre-task stimulation period**
Sclocco et al. (2019)	Model S88x, Grass Instruments, Astro-Med, Inc, West Warwick, RI, USA	Custom ergonomic electrodes, Bionik Medical Devices, Bucaramanga, Colombia	eRAVANS: 1.6 ± 2.3 mA; iRAVANS: 1.7 ± 2.4 mA; GANctrl: 1.4 ± 1.1 mA	450 μs	25	Gated to respiratory cycle (1 s per cycle)	Biphasic rectangular pulse trains	Left cymba concha; Left earlobe	8 min per condition	Brainstem fMRI (no task)	None
Sclocco et al. (2020)	UROstim, schwa-medico GmbH, Ehringshausen, Germany	Custom ergonomic electrodes, Bionik Medical Devices, Bucaramanga, Colombia	2 Hz: 7.18 ± 0.95 mA; 10 Hz: 6.46 ± 1.30 mA; 25 Hz: 5.93 ± 1.21 mA; 100 Hz: 5.57 ± 1.18 mA	300 μs	2, 10, 25, 100	Gated to exhalation (1.5 s per respirator cycle)	Monophasic rectangular pulse trains	Left cymba concha	8.5 min per condition	Brainstem fMRI (no task)	None
Borges et al. (2019)	Nemos®, Cerbomed, Erlangen, Germany	Two titanium electrodes, positioned on top of a silicon earplug	*M* = 2.3 mA (*SD* = 0.08 mA)	200–300 μs	25	30-s waves of electrical stimulation alternated by 30-s breaks		Cymba conchae of the left ear	10 min	None	None
Borges et al. (2020)	Nemos®, Cerbomed, Erlangen, Germany	Two titanium electrodes, positioned on top of a silicon earplug	*M* = 2.19 mA (*SD* = 0.93)	200–300 μs	25	30-s waves of electrical stimulation alternated by 30-s breaks		Cymba conchae of the left ear	9–17 min, depending on the task	Modified Flanker task, Spatial Stroop task, Number Letter task, and Dimension Change Card Sorting task	4 min before each task
Leutzow et al. (2013)	Nihon Kohden MEB 9200	Custom^a^	8 mA	0.1 ms duration	0.5		Electrical square impulses	Right tragus		VSEP simultaneously measured before and after Total Intravenous Anesthesia (TIVA)	
Schulz-Stübner and Kehl (2011)	NMS 300; Xavant Technology, Pretoria, South Africa		6 mA		1		Stimulated at a frequency of 1 Hzfor 30 s and then a brief tetanic stimulus was applied	Left interscalene groove	30 s		
Kox et al. (2015)	Medtronic model 37022	Stimulation catheter with eight electrodes on a circular distal loop (Achieve Medtronic model 990063-20, Medtronic, Heerlen, The Netherlands)	2–10 V	1 ms	20	Continuous		C5–C7 spinal level	30 min	Continuous physio up to 8 h following stim onset and 2 days post; 10 min before LPS administration and assess temp/symptoms every 30 min for 8 h and 2 days post	
Burger et al. (2019a)	Nemos®, Cerbomed, Erlangen, Germany	Two titanium electrodes, positioned on top of a silicon earplug	0.5 mA	250 μs	25	30-s waves of electrical stimulation alternated by 30-s breaks		Cymba concha of left ear	Across 3 tasks, this current task is 15 min (30 min + more for other tasks)	Breathing Focus Task	15 min
Hyvärinen et al. (2015)	Tinnoff Inc.	Clip electrode	0.5 mA	500 μs	25		Biphasic rectangular pulse	Left tragus	6 min	MEG	1 min
Sellaro et al. (2015b)	CM02, Cerbomed, Erlangen, Germany	Two titan electrodes fastened on a gel frame	0.5 mA	200–300 μs	25	Alternated between on and off periods every 30 s		Outer auditory canal of the left ear	75 min	Flanker and CRT, mood and physio assessed 45 and 75 min post	15 min
Verkuil and Burger (2019)	Nemos®, Cerbomed, Erlangen, Germany	Two titanium electrodes, positioned on top of a silicon earplug	0.5 mA	250 μs	25	30-s waves of electrical stimulation alternated by 30-s breaks		Cymba conchae of the left ear	Across 3 tasks + 15 min	Exogenous cuing task	15 min to first task
Jacobs et al. (2015)	TENSTem dental; Schwa-medico BV, Woudenberg, The Netherlands	Ear clip using a circular electrode of 10 mm diameter connected as an anode	5.0 mA	200 μs	8			Left external acoustic meatus on the inner side of the tragus	17 min	Continuous physio data and retrieval task post-stim	Continuous, 17 min
Burger et al. (2018)	Nemos®, Cerbomed, Erlangen, Germany	Two titanium electrodes, positioned on top of a silicon earplug	0.5 mA	250 μs	25	30-s waves of electrical stimulation alternated by 30-s breaks		Concha of the left outer ear	26 min	Extinction	12 min
Finisguerra et al. (2019)	NEMOS® device (CM02 Cerbomed, Erlangen, Germany)	Two titan ear electrodes that are mounted on a gel frame	0.5 mA	200–300 μs	25	Alternated between On/Off periods of 30 s each		Cymba conchae of the left ear	60 min	IATS	15–20 min
Colzato et al. (2018b)	NEMOS®		0.5 mA	200–300 μs	25	Alternated between on and off periods every 30 s		Outer auditory canal of the left ear	50 min	Emotion Regulation Task and assessed flow at the end	20 and 50 min
Jongkees et al. (2018)	CMO2, Cerbomed, Erlangen, Germany	Titan electrodes mounted on a gel frame	0.5 mA	200–300 μs	25	Active stimulation for 30 s, followed by a break of 30 s		Outer auditory canal of left ear	45 min	SRT	15 min
Steenbergen et al. (2015)	CM02, Cerbomed, Erlangen, Germany	Two titan electrodes mounted on a gel frame	0.5 mA	200–300 μs	25	30 s, followed by a break of 30 s		Outer auditory canal of left ear	45 min	Task test phase 25 minutes into stim and HR post-stim	25 and 45 min
Sellaro et al. (2018)	NEMOS®	Two titan electrodes mounted on a gel frame	0.5 mA	200–300 μs	25	On and off periods of stimulation alternated every 30 s		Auricle with the titan electrodes placed either in contact with the concha of left ear	35 min	Emotion regulation	20 min
Colzato et al. (2018a)	NEMOS		0.5 mA	200–300 μs	25	Alternated between on and off periods every 30 s		Concha in the left ear	40 min	Creativity tasks and personality and HRV	15 min
Fischer et al. (2018)	CMO2, Cerbomed, Erlangen, Germany	Two titan electrodes mounted on a gel frame	*M* = 1.3 mA (0.4–3.3 mA)	200–300 μs	25	Continuous		Left cymba conchae	36 min	Oddball then Simon task, physio/mood/ EEG/saliva post	Simultaneous with 2 tasks, 28 min before task of interest, 36 min
Keute et al. (2019a)	Digitimer DS7 and Arduino Uno circuit board	Medical Ag/AgCl electrodes (Ambu Neuroline3), cut to a size of 4 × 4 mm and mounted on a piece of silicone at a center-to-center	3 mA	200 μs	25	Stimulation cycle of 30 s stimulation at 25 Hz, followed by a 30 s break		Cymba conchae of the left ear; anode was placed more rostral	40 min	10-min online task	30 min
		distance of 1 cm were used									
Laqua et al. (2014)	TNS SM 2 MF device (Schwa-Medico GmbH, Germany	Anode: silver disk EEG electrode 5 mm in diameter, Schuler Medizintechnik Freiburg, Germany Cathode: (Blue Sensor PECG electrode, Ambu, Germany)	Intensity of stimulation was set individually to maximal but non-painful	0.2 ms impulse duration	Changing frequency between 2 and 100 Hz		Burst-stimulation mode	Anode: bilateral cavum conchae Cathode: mastoid area of the ear	35 min (5 min adaptation + 30 min constant)		Physio measures 15, 30, 40, and 60 min after onset
Brock et al. (2017)	GammaCore; electroCore LLC; Basking Ridge, NJ, USA	Two steel contact electrodes						(1) left cervical vagal nerve and (2) to the right cervical vagus nerve	120 s to each site		Measured 90 min and 24 h after tVNS
Peng et al. (2018)	TENS200, HUATUO GmbH, Hangzhou, China	Silver plate (5 mm in diameter) and an elongated cylindrical silver stimulation electrode (8 mm in length, 3 mm in diameter)	Around 5 mA on the acupuncture points (varied individually between 4 and 8 mA)	250 μs	20		Monophasic-modified rectangle impulse	Area of the acupuncture points CO10-12 and TF4^b^ OR anterior wall of the auditory canal^c^	First stimulation period of 30 s and a break/baseline of 60 s. Four alternating stimulation and baseline sequences were performed in total		Simultaneous MRI
Warren et al. (2019, p. 20)	NEMOS®, Cerbomed, Germany		0.5 mA	200–300 μs	25	Alternating between on and off periods every 30 s		Cymba conchae region	80 min	Saliva 45 min and post-tVNS, and pupilometry 20 and post-tVNS, EEG simultaneous, stimulus discrimination 20 min after tVNS onset	20 min
Burger et al. (2016)	Nemos_, Cerbomed, Erlangen, Germany	Two titanium electrodes, positioned on top of a silicon earplug	0.5 mA		25	30-s waves of electrical stimulation alternated by 30-s breaks		Concha of the left outer ear	~3 min (8 s*20)	During extinction	10 min
Busch et al. (2013)	STV02, Cerbomed, Erlangen, Germany	Bipolar stimulation electrode	0.25 and 10 mA	250 μS	25	Continuous	Modified monophasic rectangle impulse	Left concha at the inner side of the tragus	1 h	Continuous ANS, pain assessment	20 min
Burger et al. (2019b)	CM02, Cerbomed, Erlangen, Germany	Two titan electrodes mounted on a gel frame	0.5 mA	250 μs	25	Active for 30 s, followed by a break of 30 s		Cymba concha of the left outer ear	30 min	Fear generalization and extinction	10 min
Capone et al. (2015)	Twister—EBM	Two Ag–AgCl electrodes (5 mm in diameter)	8 mA	Pulse duration = 0.3 ms	20	Trains lasting 30 s and repeated every 5 min for 60 min	Trains composed by 600 pulses (intra-train pulse frequency = 20 Hz; pulse duration = 0.3 ms)	Left external acoustic meatus at the inner side of the tragus	60 min	Cortical excitabity TMS post-tVNS	60
Sclocco et al. (2017)		Ergonomically-shaped Ag/AgCl electrodes	Low and medium 0.10 ± 0.08 mA and 0.26 ± 0.15 mA	15 ms pulse width	25	Duration of 1 s, delivered at 25 Hz during each exhalation phase of respiration.	Rectangular pulses	Left ear	2 min		Simultaneous with paced breathing and ECG
Genheimer et al. (2017)	NEMOS cerbomed GmbH (Erlangen, Germany)		1.2 (1.1) MA	250 μS	25	30 s on and 30 s off phases		Cymba concha left ear	60 min (20 min + 40 min during task)	During extinction and during entering office for extinction; assess reinstatement next day	20 min
Usichenko et al. (2017b)	DoloBravo Dual Channel Neurostimulator (MTR GmbH, Germany)	Self-manufactured electrode, sized 9 × 9 × 2.1 mm; contact surfaces of each electrode, containing the silver wires wrapped in wool, were moistened with 0.9% NaCl solution	7.6 mA (range 5.0–11.5)	Impulse duration of 200 μs	8	Continuous	Square impulses	Concha of the auricle bilateral	~6 min		Simultaneous with heat pain during fMRI and threshold assessed post-tDCS
Cha et al. (2016)	ES-420, Ito Co., Ltd., Tokyo, Japan	Ball-type electrode	2–7 mA across sites/2 days	200 μs	30			Cymba, the cavum, and the outer surface of the tragus (Right and Left)	4 min per site	N	Assessed dizziness after tVNS
Yakunina et al. (2017)	Custom-made stimulator connected with silver wires to six electrodes	99.99% pure silver (four stimulation and two reference electrodes)	The stimulation intensities at electrodes A, B, C, and D ranged from 0.2–1.8 mA with means ± SD of 0.77 ± 0.42, 0.81 ± 0.48, 0.91 ± 0.47, and 0.81 ± 0.38 mA, respectively	500 μs	25		Monophasic rectangular impulse	4 locations in the left ear: (A) inner surface of the tragus, (B) inferoposterior wall (cartilaginous part) of the ear canal, (C) cymba conchae, and (D) earlobe. The reference electrode for electrodes A, B, and C were placed at the outer surface of the tragus, whereas the reference electrode for electrode D (sham) was placed at the backside of the earlobe	Each location was stimulated in two runs with 30 s of stimulation followed by 1 min of rest; this cycle was repeated four times in a run. Each subject underwent eight 6-min fMRI runs total, with up to 90 s of rest in between run		Simultaneous MRI
Clancy et al. (2014)	Transcutaneous Electrical Nerve Stimulation (TENS) device (V-TENS Plus, Body Clock Health Care Ltd, UK)	Modified surface electrodes	Level of sensory threshold (10–50 mA)	200 μs	30	Continuous		Inner and outer surface of the tragus of the ear	15 min		Simultaneous physio and over 15 min following recording
Antonino et al. (2017)	(TENS) device consisted of a small stimulation unit (V-TENS Plus, Body	Surface electrodes bilaterally placed	45 ± 1 mA	200 μs	30	Continuous		Inner and outer surface of the tragus	15 min		Simultaneous physio and during 10 min period post-tVNS
	Clock Health Care Ltd, UK)										
Lamb et al. (2017)		Ag/AgCl disk electrode	80% of comfort threshold of 5.6 mA (range 3–11.3 mA)	100 μS	20		Alternating polarity pulse	Left external auditory meatus and the posterior face of the left tragus		Startle blink and ANS tests	Simultaneous
Lerman et al. (2016)		Two stainless steel contact surfaces and conductive gel	M intensity ranged from 21.3 to 22.59 across the 6 stimulations		25		5-kHz sine wave series that occurred for 1 ms and repeated every 40 ms	Under the angle of the mandible, lateral to the trachea and medial to the sternocleidomastoid (right then left ear 3 times each)	2 min (90 s with 30 s ramp up	*N*	Blood draw 90 min after first stim and next day
Badran et al. (2018b)	Digitimer DS7a	Custom developed round, unipolar stimulation Ag/AgCl electrodes 1 cm in diameter, affixed to the 3D-printed clamps using cyanoacrylate	Mean ± SD: 3.14 ± 0.99 mA	500 μs	25	Three stimulation “on” periods were modeled (onset times: 30, 150, 270 s; duration 60 s)	Monophasic square waves	Left tragus	5.5 min (270 s onset time + 60 s)		Simultaneous MRI
Frøkjaer et al. (2016)	NEMOS, Cerbomed, Erlangen, Germany	Bipolar stimulation electrode	0.1–10 mA readjusted throughout experiment; mean ranged from 1.07 mA to 1.46 across time points	250 μs	30			Left concha	60 min	Cardiac derived parameters obtained at baseline and after 10, 20, and 30 min of tVNS; Quantitative sensory testing assessed at baseline and after 10 and 25 min of tVNS; Conditioned pain modulation Assessment was performed after 40 min of tVNS; Drink test for assessment of	15 min (deep breathing)
										gastroduodenal motility performed after 50 min of tVNS	
Garcia et al. (2017)	S88X GRASS stimulator, Astro-Med, Inc, West Warwick, RI	8 mm diameter, Astro-Med, Inc, West Warwick, RI	Exhalatory-gated taVNS (eRAVANS) mean ± SD: 1.22 ± 1.33 mA Inhalatory-gated taVNS (iRAVANS) mean ± SD: 0.85 ± 1.07 mA	450 μs	30	Pulse train duration of 0.5 s gated, with 0.5-s delay, after peak inhalation (i.e., during exhalation, for eRAVANS) or after peak exhalation (i.e. during inhalation, for iRAVANS)	Biphasic rectangular pulses	Auricle of the left ear [(1) the cymba concha and (2) the slope between the antihelix and cavum concha]	360 s		Airpuff stimulation was applied over the right supraorbital region of the forehead in fMRI scans pre- and post-stimulation
Rufener et al. (2018)	NEMOS tVNS device (Cerbomed, Erlangen, Germany)		0.5 mA	250 μs	25	Alternating on/off phases of 30 s		Concha cymbae of the left ear	100.5 min	Oddball task	90 min
Burger et al. (2017)	NEMOS® stimulator unit (Cerbomed, Erlangen, Germany)		0.5 mA	250 μs	25	Each CS was presented for 30 s, followed by a 40 s inter trial interval (ITI). Stimulation (sham) with the tVNS device occurred concurrently with each CS for 30 s	Monophasic square wave pulses	Concha of the left ear	Twenty unreinforced presentations of the CS+ and CS–	Fear extinction	Simultaneous with extinction task; Assessed retention 24 h later
Stavrakis et al. (2015)	Grass S88 stimulator	Flat metal clip	50% below threshold for slowing sinus rate of 39.8 ± 25.7 V	1-ms duration	20	Continuous	Square wave	Right ear, tragus cathode, earlobe anode	1 h		Post-tVNS blood draw and atrial fibrillation induction
Yu et al. (2017)	S20, Jinjiang, Chengdu City, China		50% below threshold for slowing sinus rate of 5.8 ± 3.1 mA	1-ms duration	20	Duty cycle of 5 s on and 5 s off		Right Tragus	155 ± 6 min	Holter Recording	24 h recording post tDCS and daily follow ups for 1 week
Paleczny et al. (2019)	IMER Systems, Wroclaw, Poland	Custom-made electrode	Mean amplitude= 722 ± 92 μA	1,000 μs/phase	25	Continuous or Synchronizing the stimulation with the inspiratory or expiratory phase	Rectangular, biphasic, symmetrical pulses (1,000 μs/phase, interphase interval 30 μs)	Medial of the tragus at the entry of the acoustic meatus	2 min each		Simultaneous physio
Fallgatter et al. (2003)	Two conventional bipolar electrode wires were soldered to single-sided copper claddings upon epoxy resin; At the output side two very flexible fine copper stranded wires ~10 cm in length and with a diameter of 0.05 mm, cut from a radio coil, were soldered to the copper claddings	Epoxy resin (dimension about 1 × 1 cm)	8 mA	0.1 ms duration		Interstimulus interval of 2 s	Electrical square impulses	Cathode of this bipolar stimulation electrode was placed at the inner side of the tragus at the outer ventral edge of the meatus acusticus externus. The anode was also placed at the inner side of the tragus 5 mm more distal. Alternative stimulation sites at the right ear outside the innervation of the auricular branch were tested in the single subject (lobulus auriculae, the scapha, the crus antihelices superior and the top of the helix). The distance between cathode and anode was always kept at 5 mm			Simultaneous VSEP
Polak et al. (2009)	Two conventional bipolar electrode wires soldered to single-sided copper claddings at the input side. At the output side, two very flexible fine copper stranded wires were soldered to the copper claddings. The other end of these wires was fixed to the skin with GRASS paste	Stimulation electrode was a piece of epoxy resin with two conventional bipolar electrode wires soldered to single-sided copper claddings at the input side	5, 8, and 10 mA in randomized sequence	0.1 ms duration		Interstimulus interval of 2 s	Electrical square impulses	Cathode of the bipolar stimulation electrode was placed at the inner side of the tragus at the outer ventral edge of the internal auditory meatus, the anode 5 mm away right and left ear			Simultaneous VSEP
Lewine et al. (2019)	gammaCore device		12–20 V		25	Two 120-s long bursts of stimulation applied over a 5 min period	5 kHz sine-wave stimulus for 1 ms	Left carotid sheath	5 min	EEG	15 min after active or sham tcVNS, (3) 120 min after stimulation, and (4) 240 min after stimulation
Napadow et al. (2012)	Cefar Acus II (Cefar Medical, Lund, Sweden)	0.20 × 1.5 mm modified press-tack electrodes (DBC, Korea and Vinco, China)	RAVANS [*M* (*SD*) = 0.43 (0.25 mA)]	450 μS	30	0.5 s and was gated to the exhalation phase of respiration	Rectangular pulses	(1) the cymba concha (+) and (2) the slope between the antihelix and cavum concha (–)	30 min	Deep-tissue pain intensity	15 and 30 min and 14 min after tVNS onset
Ventura-Bort et al. (2018)	CMO2, Cerbomed, Erlangen, Germany	Two titan electrodes mounted on a gel frame	*M* = 1.3 mA (0.4–3.3 mA)	200–300 μs	25	Continuous		Left cymba conchae	35 min	Oddball and Simon task	0 min; simultaneous
De Couck et al. (2017)	Cerbomed, Germany	Double ball point electrodes	Mean intensity of 0.7 mA in study 1 and 1 mA in study 2	250 μs	25	Alternating pulse series of 30 s duration followed by 30 s stimulation pause	Rectangular pulses	Cymba conchae area of the outer ear; Left ear and right ear in Study 1 and Right ear in Study 2	10 min each in Study 1 and 1 h in Study 2	HRV	Study 1: simultaneous HRV Study 2: HRV was measured during the first 5 min, between minutes 30–35 and between minutes 55–60 of the 1 h stimulation
Janner et al. (2018)	Transcutaneous electrical nerve stimulation device PuntoBravo (Medizintechnik Rostock GmbH, Rostock, Germany)	Self-manufactured electrodes; electrodes' contact surfaces, wrapped in wool, were moistened with 0.9% sodium chloride solution	*M* (*SD*) = 6.8 mA (1.3) for left ear and *M* (*SD*) = 8.3 mA (3.9) for right ear	200 μs	Mixed frequency pattern of 100/2 Hz	9 impulses with a frequency of 100 Hz emitted twice per second	Electrical square impulses	Bilateral cymbas conchae	25 min	Heat stimulation	20 min heat stimulation starts, physio and anxiety assessed 20 and 25 min after tVNS onset
Beste et al. (2016)	CM02, Cerbomed, Erlangen, Germany).	Two titan electrodes mounted on a gel frame	0.5 mA	200–300 μs	25	Active for 30 s, followed by a break of 30 s		Left inner ear	60 min	Backward Inhibition Task	Simultaneous
Hong et al. (2019)	Transcutaneous, bipolar stimulation probe (Stimulationssonde 522,015, Inomed)	Bipolar stimulation probe	10 mA	250 μs	25			Cymba conchae of the right ear	10 min		Simultaneous physio and blood draw 1 and 3 h post-tVNS
Colzato et al. (2017)	NEMOS® tVNS	Two titan electrodes mounted on a gel frame	0.5 mA	200–300 μs	25	Alternated between on and off periods every 30 sec		Outer auditory canal of left ear	35 min	Reading the Mind in the Eyes Test and physio	20 min start Reading the Mind in the Eyes Test and physio 20 and 35 min after tVNS onset
Gancheva et al. (2018)	Cerbomed NEMOS® (Cerbomed, GmbH, Erlangen, Germany)		0.6–1.4 mA for the taVNS cymba conchae condition (0.9 ± 0.1 mA, mean ± SEM)	0.25 ms duration	25	Continuous	Continuous biphasic square pulses	Cymba conchae of the left external ear	14 min	Physio	Simultaneous physio and up to 2 h post-tVNS
Kraus et al. (2013)	Voltage source (Digitimer Type DS7A, serial D127A)	MRI compatible silver plate (5 mm in diameter)	Mean stimulation intensity in the active group was 32.6 V (min 14 V, max 57 V, *SD* 13.4)	20 ms	8	Constant		Left external acoustic meatus on the inner side of the tragus	Four stimulation periods of 30 s were applied, each followed by a resting period of 1 min	*N*	Simultaneous MRI
Tobaldini et al. (2019)	TENS device (NEMOS® Cerbomed, Erlangen, Germany)	Surface electrodes	1–6 mA	200 ms	25	Continuous		Left cymba conchae of the external ear	25 min	Physio	Simultaneous physio
Yakunina et al. (2018)	Custom-made stimulator (Yakunina et al., 2017)		0.1 mA weaker than the intensity corresponding to the pain threshold means (*SD*)= 0.71 (0.43) for tragus and means (*SD*)= 0.80 (0.47) for the concha	500 μs	25	Each location was stimulated in two runs with 30 s of stimulation followed by 30 s of rest; this cycle was repeated five times in a run. Each subject underwent a total of six 5-min fMRI runs, with up to 90 s of rest between run	Monophasic rectangular impulse	Inner tragus and cymba conchae of the left ear	25 min runs per location	MRI	Simultaneous MRI
Kraus et al. (2007)	EMP2 Expert, schwa-medico GmbH, Ehringshausen, Germany	Silver plate (5 mm in diameter)	Mean intensity for LOW was 4.0 mA (SD 1.0) while for HIGH it was 5.0 mA (*SD* 1.0)	20 ms	8	Three stimulation sequences were applied, each of which consisted of a stimulation period of 30 s, followed by a resting period of 2 min. The first two sequences were performed with low stimulation intensity (LOW) and the last one with high intensity (HIGH)		Left external acoustic meatus on the inner side of the tragus	30 s	MRI	Simultaneous MRI
Keute et al. (2018)	Medical stimulation device (Digitimer DS7, UK)	Two conventional neurostimulation electrodes Ambu Neuroline, DK 4 × 4 mm	8 mA, if tolerable for the subject, and else individually adjusted below pain threshold 5.9 ± 1.6 mA	200 μs	25	Trains of 30 s, each followed by 30 s without stimulation	Monophasic square pulses	Cymba conchae of the left ear; anode being more rostral	25 min	Negative compatibility effect (NCE) task and EEG	25 min; not simultaneous
Sellaro et al. (2015a)	CM02, Cerbomed, Erlangen, Germany	Two titan electrodes fastened on a gel frame	0.5 mA	200–300 μs	25	On/Off periods every 30 s		Outer auditory canal of the left ear	30 min	Measured mood/physio data and started cyberball 20 min into stimulation and at the end of stim	~20 and 30 min
Dietrich et al. (2008)	Stand-alone electrical nerve stimulator connected with carbon fiber wires to an acrylic electrode array housing a sterling silver stimulation electrode and a reference electrode	Sterling silver stimulation electrode and a reference electrode; The array was attached to the skin with an adhesive tape	Varied individually between 4 and 8 mA	250 μs.	25	The experiment lasted 700 s and was started with a baseline lasting 100 s. This was followed by a first stimulation period of 50 s and a break/baseline of 100 s. Four alternating	Monophasic-modified rectangle impulse	Inner side of the left tragus	700 s	Simultaneous MRI	Simultaneous MRI
						stimulation and baseline sequences were performed					
Frangos and Komisaruk (2017)	Hand-held battery-operated stimulation device	A pair of non-ferromagnetic stainless steel surface electrodes (1 cm diameter)	23.9 ± 12.3 V	200 μs (1/5,000 Hz = pw in s)	25	1-ms duration bursts of 5 sinusoidal wave pulses; continuous		Right antero-lateral surface of the neck	2 min	MRI	Simultaneous MRI and rest up to 15 min after tVNS offset
Frangos et al. (2015)	Cerbomed NEMOS	Two hemispheric titanium electrodes	Earlobe: 0.3–0.9 mA Cymba conchae: 0.3–0.8 mA		25	Continuous	0.25 m-duration monophasic square wave pulse	Left cymba conchae, left earlobe	7 min		
Juel et al. (2017)	Nemos® cerbomed GmbH, Erlangen, Germany	Bipolar stimulation electrode	Ranged from 0.1 to 10 mA	250 μs	30	Continuous		Left concha	60 min	Quantitative sensory testing (QST) Conditioned pain modulation (CPM) paradigm Gastroduodenal motility parameters Vagal Tone Deep Slow Breathing	15- and 30-min after tVNS onset-start DSB for 10 min; Vagal and QST 10 and 25 min after tVNS onset and CPM 40 min post tVNS onset and motility 50 min after tVNS onset
Fallgatter et al. (2005) based on Fallgatter et al. (2003)	Two conventional bipolar electrode wires soldered to single-sided copper claddings at the input side. At the output side, two very flexible fine copper stranded wires (length	Stimulation electrode was a piece of epoxy resin (about 1 × 1 cm)	8 mA	0.1 ms duration		0.1 ms duration, the interstimulus interval was 2 s	Electrical square impulses	Cathode of this bipolar stimulation electrode was placed at the inner side of the tragus at the outer ventral edge of the internal auditory meatus. The anode was placed 5 mm		Vagus sensory evoked potential (VSEP)	Concurrent VSEP
	~10 cm, diameter (0.05 mm) were soldered to the copper claddings. The other end of these wires was fixed to the skin with a very small amount of Grass paste							more distal at the inner side of the tragus			
Tran et al. (2019)	Transcutaneous electrical nerve stimulation (TENS) unit	Ear clip electrode (Parasym device, Parasym Health, Inc., London, UK)	1 mA below the discomfort threshold	200 μs	20			Tragus of the ear	1 h	ECG	HRV after 55 min of tVNS and Echocardiography 40 min after tVNS onset
Szeska et al. (2020)	CMO2, Cerbomed, Erlangen, Germany	Two titan electrodes mounted on a gel frame	Active tVNS: average 2.28 mA Sham: average 2.53 mA	200–300 μs	25 Hz	30 s on, 30 s off		Active tVNS: cymba conchae Sham: center of earlobe	8 min	Multiple-day single-cue fear conditioning and extinction paradigm	3 min
Giraudier et al. (2020)	CMO2, Cerbomed, Erlangen, Germany	Two titan electrodes mounted on a gel frame	Active tVNS: 0.5–3.5 mA, average 1.48 Sham: 0.5–2.5 mA, average 1.31	200–300 μs	25 Hz	30 s on, 30 s off		Left cymba conchae	23 min	Lexical decision task and recognition memory task	5 min; simultaneous; 5 min. (post)
Neuser et al. (2019)	NEMOS, Cerbomed GmbH, Erlangen, Germany	Titanum electrode	Active tVNS: 0.2–3.1 mA Sham: 0.5–3.1 mA *N* = 81 *N* = 41 completed task during left side taVNS *N* = 40 completed task during right-sided taVNS		25 Hz	30 s on, 30 s off	Biphasic impulse frequency	Active tVNS: cymba conchae Sham: earlobe			
Bretherton et al. (2019) Study 1, 2	TENS machine (V-TENS Plus, Body Clock Health Care Ltd, United Kingdom)	Auricular electrode clips attached on the inner and outer surface of the tragus of the ear (Auricular Clips, Body Clock Health Care Ltd, UK)	2–4 mA	200 μs	30 Hz			Inner and outer surface of the tragus of the ear (Auricular Clips, Body Clock Health Care Ltd, UK)	15 min		
(Teckentrup et al., 2020)	NEMOS, Cerbomed, Erlangen, Germany		Individually adapted stimulus intensity (see Frangos et al., 2015)		25 Hz	30 s on, 30 s off	Biphasic impulse frequency	taVNS: left cymba conchae Sham: left earlobe	30 min		
Zhang et al. (2019)	MRI compatible Electronic Acupuncture Treatment Instrument (SDZII, Huatuo, Suzhou, China)		1.5–3 mA	0.2 ms	1 Hz	Continuous wave 20 s on vs. 30 or 20 s off block design of intermittent taVNS		taVNS: left cymba conchae Sham: left tail of the helix	13 min		

a *Electrode consisting of two stainless steel straps, wrapped with wool fiber and stapled to a 9 × 9 mm piece of silicon rubber*.

b *Silver plate was placed in the left ear triangular fossa; the cylindrical electrode was placed in the left cymba concha*.

c *Plate electrode was placed in the left external acoustic meatus on the inner side of the tragus; the cylindrical electrode was placed on the left lower limb (left middle shank)*.

## Proposed Checklist for Minimum Reporting Items

**Figure 2:**
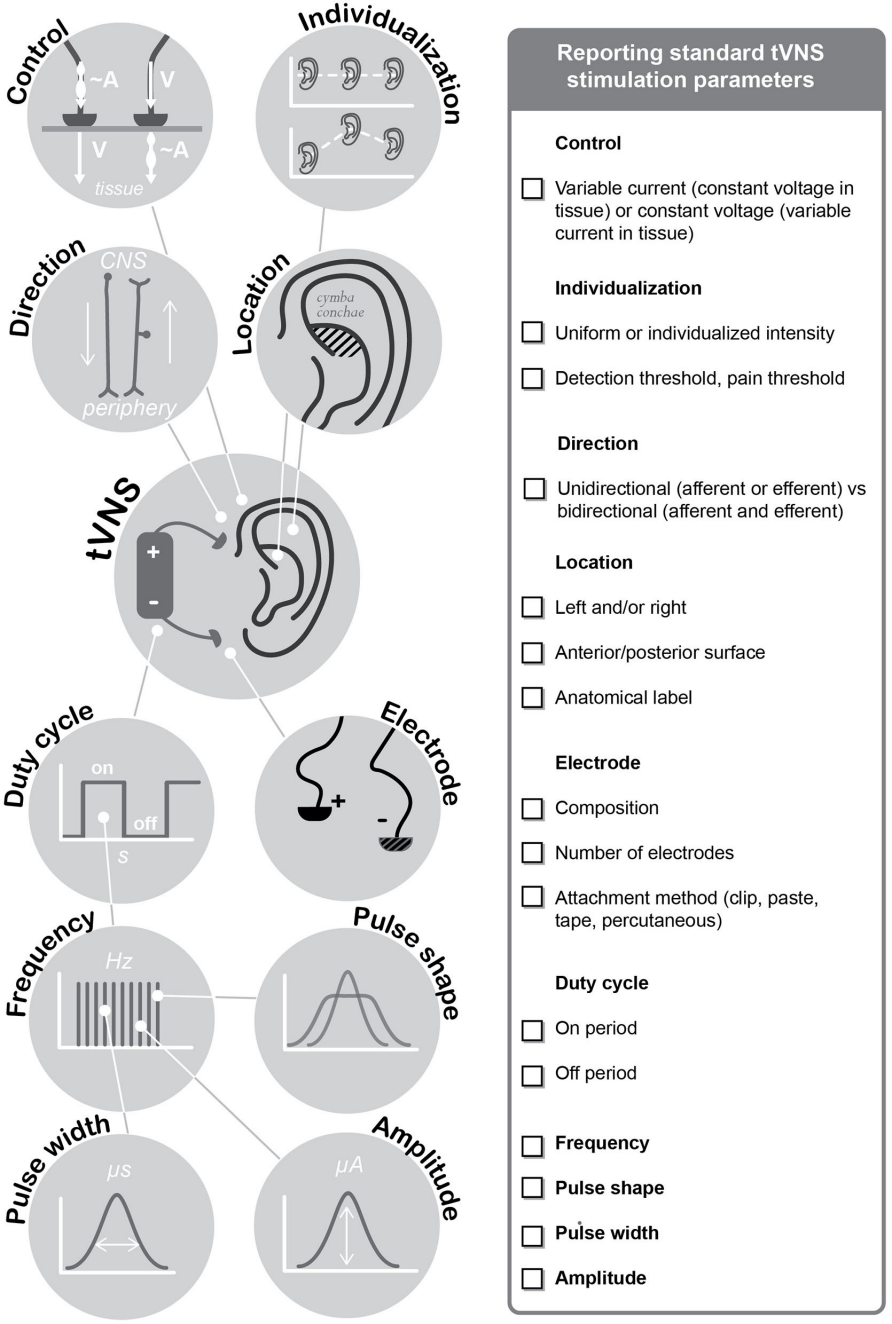
Minimum Reporting Standards for Research on Transcutaneous Vagus Nerve Stimulation (Version 2020).

Based on the review of the existing literature, we propose a set of minimum reporting items for tVNS publications in Table 3. Important to note, these are not suggested to replace existing standards or guidelines when reporting observational studies (von Elm et al., 2008) or clinical trials (Moher et al., 2001). Figure 2 provides a graphical overview of the specific tVNS reporting items.

**Table 3:** Minimum reporting standards.

**Acute/short-term stimulation**	**Long-term stimulation**
**Device level**	
– Manufacturer/name/version/edition (if applicable)	
– Regulatory aspects (CE certification, FDA compliance etc.)	
**Design level**	
– General study design (e.g., randomized controlled) – Between- vs. within-subject design (if applicable) – Blinding of subjects, assessors, and statisticians – Intended and actual session duration (min) – Pre-stimulation period (i.e., time before task/segment of interest) – Time of day (circadian influence) – Manipulation check (in sham-controlled designs) – Type of sham control (if applicable)	– General study design (e.g., randomized controlled) – Between- vs. within-subject design (if applicable) – Blinding of subjects, assessors, and statisticians – Intended and actual daily dose/total duration of intervention – Time of day of stimulation (i.e., free vs. instructed) – Protocol compliance monitoring and completer definition
**Stimulation level (for active and sham stimulation, if applicable)**	
– Stimulation site (specify anatomic location and steps in preparation) (e.g., using an alcohol wipe)	
– Electrode composition and set-up	
– Current intensity (mA)	
– Pulse width (μs)	
– Frequency (Hz)	
– Duty cycle (s)	
– Parameter descriptions: Constant current or voltage, current or voltage intensity (mA or V), pulse width, frequency, duty cycle (ON/OFF time)	
– Waveform descriptions: uni- or bi-directional, anode/cathode placement	
– Pulse shape and burst/non-burst stimulation	
– Voltage (mV) in case of voltage-controlled stimulation	
**Subject level**	
– Inclusion/exclusion criteria	
– Mean age and age range of sample	
– Sex distribution/ethnicity	
– Assessment of confounding variables	
– Prior knowledge of vagal innervation of the ear by the participant	
**Adverse events**	
– Detailed reporting on methods to assess adverse events	
– Transparent reporting on any (serious) adverse events	

In regards to stimulation level reporting, our general guidance (consistent with recommended reporting practices for other techniques, e.g., Woods et al., 2016; Bikson et al., 2019) is to fully describe the dose and any further details of electrode design that may impact tolerability. As with other reporting items, how and what details should be reported is guided by the principle of reproducibility. Dose is defined as all parameters of the device (hardware and programming) that govern the pattern of current flow through the body including to the nominal nerve target (Peterchev et al., 2012). For electrical stimulation dose encompasses: (1) all aspects of the stimulation waveform (e.g., pulse shape such as square, frequency); (2) details of electrode contact with the skin (e.g., size, shape, location). Factors that go into selecting dose, on a trial or subject basis (such as titration to sensation) are critical to report, but the actual dose applied should also be reported (Peterchev et al., 2012). It is important that complete details of dose be reported, not simply those aspects of dose the investigators think are important to outcomes (or important to mention). It is also important to recognize that referencing a technique by a name of classification does not fully describe dose since the same name may be used to describe different protocols (Guleyupoglu et al., 2013; Bikson et al., 2019). Nor is it sufficient to describe dose by referring to prior publications when those publications did not fully describe dose, when those prior works described a range of approaches broader than tested in the present study, or when any modifications (even incremental) were made. Finally, careful attention should be paid to the use of nomenclature (Bikson et al., 2019) that is not definitive in describing the dose (e.g., unipolar, anodal), may apply to different aspects of the dose (e.g., pulse duty cycle or train duty cycle) or mis-applying terminology (biphasic vs. bipolar).

Details of electrode design, preparation and application that are no genuine part of dose are likewise critical to allow consistent dosing. For example, the critical interface is the contact *surface* between the tissue and electrolyte e.g., hydrogel, paste (for a non-invasive electrode), or metal (for a percutaneous electrode). This needs to be described for every electrode, including electrodes that are considered less important for outcomes (e.g., so called “return” or “reference” electrodes). Other aspects of the electrode, such as materials and thickness, are equally important for reproducibility, including, for example, electrochemical stability (Merrill et al., 2005) and tolerability (Minhas et al., 2010; Khadka et al., 2018).

## Discussion

Having proposed a set of reporting standards, we will now address some of the outstanding issues, which in our view, future tVNS studies have to objectively and systematically address. These issues have all been examined in previous studies to a greater or lesser extent, but given the lack of reporting standards, no definite conclusions can yet be drawn. It is our hope that having provided these standards, clear answers will become apparent in the years to come. Here we will subsequently discuss issues related to safety, confounding, stimulation parameters, underlying physiology including studies on biomarkers and translational studies.

### Safety and Tolerability

In line with our recommendations of providing standardized information on stimulation parameters etc., we encourage the standardized reporting of adverse events as suggested by Redgrave et al. (2018). A systematic literature review on the safety and tolerability of tVNS has evaluated 51 studies, independent of the area of application (Redgrave et al., 2018). The authors report that the most prevalent side effect was local skin irritation from electrode placement, occurring in about 18% of included subjects following long-term stimulation. Nevertheless, it is important to note that 89 studies were not included in this review as these studies had not reported safety or tolerability data and when approached the authors didn't respond to a formal request to provide data.

### Potential Confounding Variables

Alongside transparent reporting of stimulation parameters and adverse events, important confounding variables need to be considered and reported. The inter-individual variability in the neurophysiological and behavioral response to tVNS is high and the reasons for this are poorly understood. A diverse array of factors including, but not limited to age and comorbidities, subjects' ear and tissue morphology and innervation, neurotransmitter balances and brain state, may contribute to inter-individual differences in tVNS response. Based on studies using tVNS, iVNS, and other electrical stimulation techniques, we suggest that investigators consider the following variables that can influence the responsiveness to tVNS and can confound the results in their studies.

#### Age

Increasing age affects both parasympathetic and sympathetic activity (e.g., Kuo et al., 1999). For example, age is associated with marked changes at hormonal level, which in turn affect acetylcholine-mediated parasympathetic autonomic activity, which is affected by tVNS (Moodithaya and Avadhany, 2012; Krause and Cohen Kadosh, 2014). Furthermore, sensitivity to electrical transcutaneous stimulation is lower in older age-groups (Kemp et al., 2014).

#### Sex

In animal studies VNS has greater effects in females, probably because of the effect of oestrogens to the muscarinic acetylcholine in the central nervous system (Du et al., 1994). Similar effects should be expected in human subjects due to both hormonal levels and the gender- and age-dependent differences in the functions of the autonomic nervous system (Koenig and Thayer, 2016; Koenig et al., 2017). Differences in the neuronal pathways and neuronal sensitivity may exist and therefore affect response to tVNS (De Couck et al., 2017; Janner et al., 2018).

#### Medical Conditions

Neurotransmitter levels may differ between individuals according to specific medication intake and medical condition. This was shown to cause research subjects to respond differently to stimulation due to a certain dose-response relationship that interacts with initial neurotransmitter levels (Ziemann et al., 2002; Falkenberg et al., 2012). Therefore, to avoid confounds in experiments, we recommend to control for (or exclude) individuals with psychological or psychiatric conditions (e.g., Homma et al., 1993; Salman, 2015) and medication use that affects neurotransmitter systems (unless those study populations are directly relevant to the research question).

#### Ear and Tissue Anatomy

Different ear sizes and skin properties, such as impedance, water content, structure, and subcutaneous fat thickness as well as auricular anatomy of the vagus innervation may cause different current distributions and require different current strengths to achieve the same current flow (Maffiuletti et al., 2008; Cakmak, 2019). Consequently, physiological and behavioral effects may vary.

#### Time of the Day /Different State

The brain does not always respond stereotypically to stimulation, as response may depend on the current state of activity (Silvanto et al., 2008), level of fatigue, wakefulness, attention, or mood (Sztajzel et al., 2008; Steenbergen et al., 2020). Controlling for brain state, for instance, by employing a focused behavioral task, or applying stimulation only during a particular brain state, e.g., based on patterns of electroencephalographic (EEG) activity (Brázdil et al., 2019), may potentially improve responsiveness. This may also extend to physiological states in general, such as respiratory phase (Napadow et al., 2012; Garcia et al., 2017; Sclocco et al., 2019).

#### Adherence

Especially in neuropsychiatric populations, adherence must be controlled. Dependent on the population non-adherence rates up to 50% (Perkins, 2002) have been reported from pharmaceutical trials and it must be assumed that the same numbers will occur. Such non-adherence rates have e.g., reported for the tVNS schizophrenia trial (Hasan et al., 2015) and the adherence should be recorded and analyzed in all future tVNS trials.

#### Control Condition

Control condition is tVNS tested against sham stimulation (actual stimulation of the earlobe, for example), or no stimulation. Further, authors should report on placebo/expectations effects and which attempts were made to control for this influence. Very recently, problems with the wrongful placement of electrodes in sham-stimulation, in particular the possibility to stimulate muscle zones with potential effects, have been discussed (Cakmak et al., 2017; Liugan et al., 2018).

In future, it is important that researchers are aware of sources of variability that may affect tVNS response, especially in studies using heterogeneous populations, and that they select their desired research population with caution. Furthermore, tracking potential confounds may allow the investigators to control for them in the analysis and to understand outliers within the data. This approach may help to understand factors explaining heterogeneity in the efficacy and response to tVNS.

### Left or Right? A Question of Laterality in VNS Targeting

Anecdotally during the development of iVNS, theoretical concerns emerged regarding cardiac safety when implanting electrodes on the right cervical VN in comparison to the left. This theory was only explored in one iVNS trial exploring both left and right iVNS for chronic heart failure which demonstrated equal safety profiles (Premchand et al., 2014). Animal studies suggest that right sided iVNS has stronger cardiac effects (Ng et al., 2001; Yoo et al., 2016). Due to this uncertainty, an important constraint when applying taVNS is the choice of the ear side during stimulation. Individual stimuli delivered to the right cervical VN have two-fold inhibition effects on heart beating cycle, compared to identical stimuli delivered to the left nerve (Brown and Eccles, 1934). The reason is that efferent vagal fibers affecting the sinoatrial node of the heart are thought to be right-lateralized (Nemeroff et al., 2006). Studies in rats have shown that vagal fibers originating in the right dorsal nucleus and the right ambiguous nucleus further inert the region of the syno-atrial nodule, while the fibers of the left dorsal motor nucleus and the projected ambiguous further inert into the atrioventricular nodule region (Brack et al., 2004). Despite the possible side effects of right sided vagal stimulation, a possible treatment for heart failure has been developed using a tcVNS device, measuring the heart rate, that shuts down when bradycardia is detected (De Ferrari and Schwartz, 2011). However, for reasons outlined above, and possibly because a clinical trial showed no arrhythmic effects of tVNS when stimulating the left VN (Kreuzer et al., 2012), taVNS is almost exclusively applied to the left ear. Yet, these concerns have been challenged (Chen et al., 2015). For instance, studies in rodent models have not shown deleterious cardiac side effects (Krahl et al., 2003, also see Ay et al., 2011; He et al., 2013b). A study in healthy human participants has shown that taVNS can be applied to the right ear without associated cardiac side effects (De Couck et al., 2017). Similarly, studies in patients with chronic heart failure (Premchand et al., 2014; Wang et al., 2015b) did not report cardiac side effects suggesting that bilateral or right-lateralized taVNS is not associated with an excess rate of adverse effects. Furthermore, varying the intensity of taVNS has been shown not to impact on cardiac vagal activity in healthy adults (Borges et al., 2019). Critically, to the best of our knowledge, no systematic safety studies to date have directly compared stimulation sites and duration of stimulation to examine possible cardiac adverse effects.

The possibility of safely stimulating both, the left and right VN simultaneously, is of interest. In terms of using tVNS to increase noradrenaline release, it is plausible to suggest that bilateral stimulation may improve efficacy. Animal experimental data suggest a very wide spectrum of effects, critically dependent on stimulation parameters as well as on the duration of stimuli trains (Levy et al., 1969; Slenter et al., 1984) phase-locking the heart beat to the vagal stimuli (Jalife et al., 1983) through the interaction of neural and muscular reflexes (Brooks and Lange, 1977). It has been shown that tVNS activates brain regions with ipsi and against lateral differences—such as the nucleus of the solitary tract, the amygdala or the nucleus accumbens (Frangos et al., 2015). LC projections to the cortex are mainly ipsilateral (Aston-Jones and Waterhouse, 2016), and noradrenaline levels are increased in both hemispheres after iVNS in rats. It has also been shown that depending on the currents applied (iVNS), different neuronal populations are recruited, and moreover that noradrenaline release in different target areas is also current-dependent (Roosevelt et al., 2006). Thus, hypothetical by stimulating both ears simultaneously, a summation effect could potentially be attained to reach the desired effects (also see Clancy et al., 2014). This idea should be objectively evaluated in the future, since pain threshold in some patients can be as low as 0.5 mA when auricular stimulation is carried out, and therapeutic effects could require higher stimulation currents (>1.0 mA) (Yakunina et al., 2017).

### Current-Controlled vs. Voltage-Controlled Stimulation

In this iteration of the consensus, we would like to particularly focus on one particular technical aspect, namely the proper reporting on whether current-controlled or voltage-controlled stimulation is used. In principle current or voltage control settings can be used for tVNS; however, effects of and on the electrode/tissue boundary have to be accounted for (Merrill et al., 2005; Kaniusas et al., 2019a). As is generally the case in neuromodulation (Butson and McIntyre, 2005; Merrill et al., 2005; Vargas Luna et al., 2013), the current-controlled reliably defines the current in the body (e.g., excitable auricular tissue) independent of the highly variable electrode/tissue boundary. However, in the case of voltage-controlled tVNS, the resulting current in the tissue depends strongly on the electrode-skin boundary properties which then influence the resulting stimulation efficiency. The impact of current-controlled vs. voltage-control on the effectiveness will depend on multiple factors including electrode design. For instance, needle electrodes (for example in percutaneous tVNS) act typically as polarizable electrodes so that the boundary is predominantly capacitive, whereas surface electrodes (for example in taVNS) can act as non-polarizable electrodes with a predominantly resistive boundary. One theoretical concern with current-controlled stimulation is that conditions of unexpected high impedance at the electrode-skin interface will result in an associated increase in stimulator output voltage (i.e., needed to overcome this resistance in providing a prescribed current). The maximum voltage is limited by stimulator output compliance voltage. In a situation where the impedance suddenly changes, which can result from the electrode becoming displaced or (partially) detached and then reattached to the skin, a current controlled device may transiently produce a current above the target level (as its internal circuit adjust to the lower impedance load), which in turn can result in an unpleasant shock. This can be readily addressed with robust and motion-free application of current electrodes (e.g., ear clip electrodes, reliable adhesive electrodes), protocols that are cognisant of factors such as when stimulators are powered (Badran et al., 2019), or stimulators that are designed with a rapid accommodation time.

In the case of the voltage application, the polarization voltage is limited by the applied voltage. In addition, any potential detachment of the voltage electrode leads to an even reduced polarization voltage and thus reduced risks of unwanted transients. Consequently, voltage-controlled stimulation may be limited by current changes in situations where electrode-skin contact is not reliable. In addition to adverse events that can result from current flow through the body (e.g., pricking/itching), as with any electrical stimulation, adverse events may result from excessive electrochemical reactions at the electrode-electrolyte interface (Kaniusas, 2019). Specifically, if electrochemical products at the electrode-electrolyte interface reach the skin, skin irritation may ensue. Protocols to limit this include using charge-balanced waveforms (Sooksood et al., 2009, 2010), judicious selection of metal and electrolyte materials (Merrill et al., 2005; Khadka et al., 2018), minimizing total stimulation time at a given location, or ensuring the electrolyte provides sufficient separation between the metal and skin (Minhas et al., 2010).

### Empirical Evidence for the Use of Certain Stimulation Parameters

Currently, the popularity of one tVNS device, tVNS Technologies GmbH (Erlangen, Germany), led to a common yet poorly argued parametric setting. Given the lack of flexibility of this device regarding changing the parameters, a signal with a pulse width between 200 and 300 μs at 25 Hz, and a duty cycle of 30-s on, 30-s off has frequently been adopted in studies. However, other parameters have been used in research with tVNS as well, which may explain in part the heterogeneity observed in findings from studies using tVNS (Borges et al., 2019). Consequently, the lack of knowledge regarding optimal stimulation parameters can be seen as a general limitation in this research field (Borges et al., 2019; Butt et al., 2020). Despite an understanding of the importance of the various stimulation parameters in optimizing the efficacy of tVNS, dose-response studies remain scarce. Recently, Badran et al. (2018c) systematically tested the effect of three variations in pulse width and frequency, respectively, on HR and found that a pulse width of 500 μs, if combined with a frequency of 10 Hz, produced the strongest decrease in HR compared to other parameter combinations. However, as HR is the result of mixed inputs from the sympathetic and parasympathetic (vagus) nerves, the effect of tVNS on HR may not necessarily correlate with the outcome of interest (Goldberger et al., 2019). Therefore, we advocate caution when interpreting these results. Some efforts have been made to understand how changing specific stimulation parameters influences the physiological effects of tVNS. Borges et al. (2019) tested the effect of different intensities on cardiac VN activity (Malik, 1996) in three experiments. They also compared different methods to define current intensity regarding cardiac vagal activity, namely presetting the same current intensity for all study participants throughout the experiment (set method) and instructing the study participants to freely choose an intensity (free stimulation method). Cardiac vagal activity increased during tVNS when compared to resting measurement. However, this increase was not related to stimulation intensity, the method of stimulation, or whether the stimulation was active or sham. De Couck et al. (2017) investigated the effect of stimulation side (*right, left ear*, or *sham*), and session duration (10 min or 1 h) on heart rate variability (HRV). They found very specific effects related to heart rate variability components such as standard deviation of the RR intervals (SDNN) as well as low frequency (LF) and LF/high frequency (HF) ratio. However, tVNS had no effects on parameters that serve as an index of cardiac vagal activity, such as root mean square of successive differences in RR intervals (RMSSD) (Malik, 1996). Changes in the frequency domain components of HRV (LH, HF, and LF/HF ratio) were also observed with 1 h of tVNS at the right tragus (Tran et al., 2019). It was also reported that the magnitude and direction of tVNS-induced changes in LF/HF ratio is dependent on resting LF/HF ratio (Bretherton et al., 2019). The greatest effects of tVNS were observed in individuals with the lowest cardiac vagal activity at rest. Regarding stimulation location, Yakunina et al. (2017) compared the effects on brain activation of stimulation carried out at the inner tragus, inferoposterior wall of the ear canal, cymba conchae, and earlobe (sham). Among these areas, only tragus and cymba conchae stimulation activated areas thought to be part of the vagal pathway, such as the NTS. Importantly, the strongest activation of vagally innervated areas was seen during cymba conchae stimulation. These results are consistent with anatomical studies suggesting that the auricular branch of the VN innervates primarily the cymba conchae and the tragus (Peuker and Filler, 2002). Interestingly, a recent study by Sclocco et al. found that stimulation frequency also significantly modulates BOLD fMRI response in NTS, as well as other brainstem nuclei such as LC and raphe nucleus (Sclocco et al., 2020), with 100 Hz stimulation demonstrating enhanced activation in healthy adult volunteers. As anatomy is fundamental to providing effective tVNS (Badran et al., 2018a, also see Burger and Verkuil, 2018), further studies are warranted to delineate the exact anatomical basis of tVNS, in order to better guide future trials.

To summarize, the choice of stimulation parameters, mainly linked to pulse width, frequency, side and location of the stimulation, may influence effects of tVNS on both autonomic and cognitive processes. However, attempts to investigate the effects of individual tVNS stimulation parameters have primarily focussed on presumed physiological effects of tVNS rather than cognitive processes. Furthermore, despite first attempts to address the effects of parametrization, it is not clear what cognitive or autonomic processes have a parametric-specific effect, and this could explain the high heterogeneity of findings in studies using tVNS. Thus, it is time to carry out further studies that aim at understanding the parametric-specific effects of tVNS in order to optimize this tool for different applications.

### Potential Biomarkers of Effective Stimulation

The neural mechanisms mediating the effects of tVNS are still poorly understood and, consequently, no clear consensus exists about potential biomarkers that could shed light on the efficacy of tVNS in general, or those guiding a choice in stimulation parameters. In this section, we briefly summarize findings concerning potential biomarkers related to vagal activity (for a detailed review about biomarkers of tVNS, see (Burger et al., 2020a) and finish with some remarks on methodological aspects that may be relevant when assessing biomarkers in tVNS research.

#### Heart Rate Variability

Some authors have proposed that the beneficial effects of tVNS may rely on increased activity of the VN *per se* (Gidron et al., 2018). Therefore, tVNS-related changes in vagal activity—measured by vagally-mediated HRV measures (vm-HRV) (Thayer and Lane, 2000; Kuo et al., 2005) may be informative to its efficacy. Animal research has consistently found that VNS, particularly to the right VN, increases vm-HRV measures (e.g., Huang et al., 2010; Sun et al., 2013). However, the relation between iVNS and HRV measures is less clear in humans (see Burger et al., 2020a for further details). Similar to reports using iVNS, findings on the modulatory effects of tVNS on vm-HRV measures are heterogeneous. Some studies showed an increase of vm-HRV measures after tVNS (Lamb et al., 2017; Bretherton et al., 2019; Sclocco et al., 2019; Tran et al., 2019), but others showed no effects (Weise et al., 2015; Antonino et al., 2017; De Couck et al., 2017; Burger et al., 2019a,b), or showed a decrease of vm-HRV parameters in individuals with high resting vagal activity (Bretherton et al., 2019) and two other studies during both, active and sham stimulation (Borges et al., 2019, 2020). A potential limitation of vm-HRV measures as a biomarker for tVNS is that the mechanism influencing vm-HRV (i.e., efferent vagal activation) may differ from the mechanistic target of tVNS (i.e., afferent vagal activation), and little is known about the interrelation of these two vagal pathways, i.e., much is known about cervical vagal feedback loops, but not much is known regarding auricular to cervical loops.

#### Metabolic Markers of Vagal Stimulation

The VN is a key part of the autonomic nervous system and transmits information between the peripheral organs and the brain to support homeostasis (de Lartigue, 2016). Although vagal stimulation primarily targets afferent fibers, preclinical and human work points to efferent effects as well that are mediated via the brain. In animal studies, there is conclusive evidence for reduced food intake and weight loss following iVNS (Roslin and Kurian, 2001; Val-Laillet et al., 2010; Gil et al., 2011; Banni et al., 2012). In rodents, a closed-loop VNS system implanted on the stomach wall substantially reduced food intake and delayed weight gain (Yao et al., 2018) demonstrating the modulatory role of negative feedback signals. In human studies, the vital role of the VN in modulating food intake, energy metabolism, and glycemic control has been demonstrated more recently (Burneo et al., 2002; Pardo et al., 2007; Shikora et al., 2013; Ikramuddin et al., 2014; Cork, 2018). Notably, taVNS has been shown to decrease the frequency of action potentials in human gastric muscle cells (Hong et al., 2019; Teckentrup et al., 2020) suggesting that an electrogastrogram could be used to non-invasively track successful vagal stimulation. Taken together, these results highlight that stimulating vagal afferents may elicit efferent effects on key markers of energy homeostasis that could be used as a positive control outcome.

#### Noradrenergic-Related Processes and Markers

One potential mechanism by which tVNS may exert its effect is through the activation of the LC norepinephrine (LC-NE) system (Van Leusden et al., 2015; Hansen, 2019). Evidence pointing to a modulatory role of VN activity on LC-NE system activity comes from neuroimaging studies (Dietrich et al., 2008; Kraus et al., 2013; Frangos et al., 2015; Yakunina et al., 2017) and from studies relating vagal activity with physiological markers of LC-NE system activity, such as the P300 amplitude of event-related potentials (ERPs) (Murphy et al., 2011) see for review (Nieuwenhuis et al., 2005), salivary alpha amylase(sAA; Ehlert et al., 2006; Warren et al., 2017), and pupil dilation (Rajkowski, 1993; Joshi et al., 2016; Warren et al., 2017). For instance, De Taeye et al. observed that epileptic patients that responded favorably to iVNS therapy showed an increase in the P300 amplitude during VNS (De Taeye et al., 2014) see also (Neuhaus et al., 2007; Schevernels et al., 2016; Wostyn et al., 2017). In healthy participants, however, evidence for the modulatory effects of tVNS on the P300 amplitude has been mixed. Some studies found enhancing effects (Rufener et al., 2018; Ventura-Bort et al., 2018; Lewine et al., 2019), but others found no modulation of the P300 (Warren et al., 2017; Fischer et al., 2018). In terms of pupil dilation, although evidence about the relation between VNS and pupil dilatation is rather scarce, findings in animals (Bianca and Komisaruk, 2007; Mridha et al., 2019) and humans (but see Schevernels et al., 2016; Jodoin et al., 2018) seem to point to increased dilation of the pupil under active iVNS compared to no stimulation. By contrast, in four recent tVNS studies, no modulation of pupil dilation in response to the stimulation was found (Keute et al., 2019b; Warren et al., 2019; Burger et al., 2020b). Finally, recent studies have investigated the effects of tVNS on sAA levels as a potential marker of central NE release (Ehlert et al., 2006; Warren et al., 2017). Similar to P300 and pupil dilation, studies exploring tVNS effects on sAA level changes have shown inconsistent results. Some showed increased sAA levels following tVNS, but not after sham stimulation (Fischer et al., 2018; Ventura-Bort et al., 2018; Warren et al., 2019). Three recent studies did not show any sAA changes in response to tVNS (Koenig et al., 2019; Giraudier et al., 2020; D'Agostini et al. under review), Also, documented improvements of sleep quality with tVNS (Bretherton et al., 2019) are inconsistent with LC activation, which is the main brainstem nucleus that promotes arousal.

Taken together, there is currently no reliable vagal or noradrenergic biomarker of tVNS that produces replicable results across studies. It is likely that the reasons for this are multifactorial [see for a detailed discussion, (Burger et al., 2020a)]. Firstly, many studies included relatively small sample sizes, and the reported effects may have been underpowered. Secondly, baseline differences in tonic noradrenergic activation may also have an important influence on the efficacy (Murphy et al., 2011; Gee et al., 2014; van Kempen et al., 2019). Finally, stimulation settings, including stimulation sites (tragus vs. cymba concha; left vs. right ear) and parameters such as stimulation interval (30 s ON/OFF vs. continuous stimulation), intensity set-up (fixed or variable across participants), pulse widths, stimulation timing, among others, are not kept constant across experiments, impeding, to some extent, a full comparison of the results across labs. These changes might not be arbitrary, given that some of the settings may favor the efficacy of tVNS (e.g., stimulation of cymba conchae compared to the tragus (Yakunina et al., 2017); continuous vs. intermittent stimulation (Ventura-Bort et al., 2018); and long compared to short stimulation duration (Warren et al., 2019). We hope that the aforementioned standards may help overcome these challenges and improve the current knowledge about potential tVNS biomarkers.

#### Functional Neuroimaging

In comparison to HRV, pupil dilation and sAA, *functional magnetic resonance imaging fMRI* provides the possibility to confirm involvement of the central noradrenergic system by looking directly at LC and NTS activation as well as activation of possible target areas. Consequently, neuroimaging studies have tried to assess the modulatory role of VN activity on the LC-NE system activity in healthy adults (Kraus et al., 2007, 2013; Dietrich et al., 2008; Frangos et al., 2015; Yakunina et al., 2017; Badran et al., 2018b; Peng et al., 2018; Sclocco et al., 2019, 2020) and interictal migraine patients (Garcia et al., 2017; Zhang et al., 2019). The following results in functional activation are based on the comparison between real and sham stimulation at varying stimulation locations across the studies (see Table 1). Three studies that examined activation using 1.5T imaging (Dietrich et al., 2008) did not report any sham stimulation, therefore their results were based only on active stimulation compared to pre-stimulation baseline (*N* = 4). The authors found increased activation in the left LC as well as an increase in functional activation in the left thalamus (Dietrich et al., 2008). Conversely, a decrease in functional activation in limbic and temporal brain areas (*N* = 6) (Kraus et al., 2007) as well as in the LC and the NTS (*n* = 8) (Kraus et al., 2013) has also been shown. However, the reported functional activation of these three studies might have to be interpreted with caution due to the low sample sizes. Additionally, it is possible that the spatial precision afforded in data acquisition and data processing was not sufficient in these studies to reliably detect activations in LC and the NTS which are only a few millimeters wide. Furthermore, none of these studies reported information on the MRI head coil or smoothing kernel used which makes it difficult to assess Signal-to-Noise Ratio (SNR) and spatial precision of the results (Kraus et al., 2007, 2013; Dietrich et al., 2008). Studies with larger sample sizes using 3T scanners have shown *functional activation* in NTS (Frangos et al., 2015; Garcia et al., 2017; Yakunina et al., 2017; Sclocco et al., 2020), in the bilateral amygdala and left parahippocampal gyrus (Peng et al., 2018), which corresponds to the results of Frangos et al. (2015) that showed an increase in activation in the contralateral amygdala, nucleus accumbens and anterior thalamic nuclei. Moreover, a gradual increase and maximal activation in NTS during post-stimulation was observed (Frangos et al., 2015). In addition to increased NTS activation, post stimulation effects, immediately after exhalatory-gated auricular vagal afferent nerve stimulation (eRAVANS), led to increased response to trigeminal sensory afference in nucleus raphe centralis and LC (Garcia et al., 2017). Yakunina et al. (2017) showed bilateral LC and NTS activation in unsmoothed data and indicated that this was also observed during real stimulation by placing electrodes at the inner surface of the tragus. Badran et al. (2018b) were not able to replicate these effects. However, this study used lower resolution fMRI (voxel size of 3 mm3), which may explain the lack of activation observed in the NTS and LC. Some studies have also reported a *decrease in functional activation* in the bilateral hypothalamus and throughout the hippocampal formation in healthy adults (Frangos et al., 2015) as well as in the bilateral LC in interictal migraine patients (Zhang et al., 2019). This heterogeneous pattern of functional activations reported highlights once again the challenge of comparing and interpreting results from fMRI tVNS studies using different devices, electrodes, stimulation locations and session durations, as already previously reviewed (e.g., Yakunina et al., 2017; Peng et al., 2018). It is furthermore equally important to consider the varying stimulation and rest phases of study designs in different studies (e.g., 0.5 s pulse during each exhalation phase of respiration Garcia et al., 2017; 7 min on/2 min off stimulation Frangos et al., 2015; 30 s on/60 s off stimulation Yakunina et al., 2017; Peng et al., 2018), which might have affected the ability to detect functional activations in NTS and LC target areas. As there is considerable one possible focus in using tVNS fMRI to study evoked activation in NTS and other neurotransmitter source nuclei following stimulation, the fMRI studies of Yakunina et al. (2017) and Sclocco et al. (2019) used high resolution (e.g., 2.75 mm, and 1.2 mm isotropic resolution, respectively) and small Gaussian smoothing kernels (e.g., 2 mm), provide promising spatial precision in their methodological approach. Moreover, both studies compared results across various stimulation locations and observed most convincing LC activations using the left cymba conchae as an active stimulation location, which is in line with left cymba conchae being considered a good target for eliciting LC activation (Peuker and Filler, 2002). Both Yakunina et al. (2017) and Sclocco et al. (2019) provided details on co-registration methods and demonstrated sufficient spatial precision in data processing. The latter study used ultra-high-resolution fMRI at 7 Tesla with multi-band factor 2 to further increase SNR and demonstrated that exhalatory-gated tVNS enhanced NTS and LC/raphe targeting. Similar to Garcia et al. (2017), they observed increased activation in the LC as well as both dorsal and median raphe nuclei and in contrast to previous studies, they implemented short duration stimulation events (1s) extended over many minutes of time (Sclocco et al., 2019). Whilst many 3T fMRI studies may lack sufficient spatial precision to answer the question whether tVNS can target NTS and LC, recent studies suggest that larger sample sizes can also show NTS and LC response at this lower field strength (Sclocco et al., 2020), and previous 3T studies are more numerous and provide the strongest support that tVNS may indeed be a suitable tool for targeting the LC-NE system. Disorder specific brain circuits have been discussed as potential targets for tVNS in depression (Iseger et al., 2020) and tinnitus (Yakunina et al., 2018).

Taken together, when validating tVNS effects in various populations with the use of the most direct biomarker at hand for the LC-NE system, i.e., fMRI—a number of methodological considerations should be kept in mind over and above the usual need for appropriate stimulation parameters. Specifically, given that both NTS and LC span only a few millimeters, the extent of smoothing across studies should be considered. Frangos et al. (2015) pointed out the concerns of applying spatial smoothing to brainstem nuclei. Choosing a too high smoothing factor [e.g., 6 mm (Peng et al., 2018) or 8 mm (Yakunina et al., 2017)] could lead to an increased likelihood of false positives or to no observable activation in brainstem nuclei and thus, some chose to forgo smoothing brainstem data (Frangos et al., 2015; Yakunina et al., 2017). Similarly, ultra-high-resolution fMRI in the range of 1–2 mm voxel sizes also at higher field strengths seems warranted as well as customized high-precision spatial post-processing approaches optimized for the LC-NE system [see (Liu et al., 2017) for a review]. In addition to using comparable set-ups across studies, further research should also try to incorporate structural measures of the LC-NA system such as neuromelanin (NM)—sensitive magnetic resonance imaging (MRI) to anatomically identify the LC *in vivo* (Sasaki et al., 2006; Betts et al., 2017, 2019; Hämmerer et al., 2018; Priovoulos et al., 2018; Liu et al., 2019; Trujillo et al., 2019; Ye et al., 2020). Finally, the increased susceptibility of brainstem fMRI for low SNR and high physiological noise (Sclocco et al., 2018) could be counteracted by appropriate imaging paradigms as well as denoising or noise-control approaches (Brooks et al., 2013; Sclocco et al., 2018). If these recommendations are kept in mind, fMRI carries great potential as a more precise and direct tool for identifying activation in the LC-NE system using tVNS and in future may help to differentiate between tVNS responders vs. non-responders.

### Toward Circuit-Based tVNS: Translational Approaches

Despite the growing interest in tVNS and in particular taVNS in clinical applications, many human studies remain in explorative frameworks and are typically confined to indirect readouts or neuronal activity of indirect fMRI responses (Yakunina et al., 2017; Burger et al., 2020a). Besides, imaging of small pontine nuclei such as the LC, NTS, or the raphe nucleus can be challenging in humans using MRI/fMRI, even at the purely anatomical level (Betts et al., 2019). Animal experimentation, on the other hand, can employ invasive techniques that allow researchers to gain detailed insights in molecular, anatomical, and neurophysiological mechanisms involved in VNS therapy. Thus, animal models enable a systematic investigation to be undertaken not only in terms of specificity of their readouts, such as cellular activity or level of neuromodulators, but also in terms of delineating parameter space for effective stimulation.

Effectiveness of VNS stimulation can be detected either directly with high temporal resolution i.e., *in vivo* electrophysiology as well as calcium imaging or more indirectly, after stimulation, using immunostaining or mRNA probes against immediate early genes products (C-Fos, Arc, Egr1) available in several animal models (Groves et al., 2005; Manta et al., 2009; Ay et al., 2016; Hulsey et al., 2017). Activation of the NTS, LC, and raphe nucleus after VNS has also been monitored via extracellular electrophysiological recordings (Groves et al., 2005; Manta et al., 2009; Hulsey et al., 2017). Hulsey and colleagues mapped the stimulation space using LC neuron spiking activity as an output variable. Although this study was performed using invasive VNS, it was clearly shown that the application of low currents (0.1–1.2 mA) induced LC neuron firing, but higher currents (>1.2 mA) also activated neighboring Me5 neurons (Hulsey et al., 2017). This finding is of particular importance since different neuronal populations with distinct axonal projection could be potentially recruited depending on the set of stimulation parameters chosen. Also such findings can explain the broad scope of responses seen in human studies under sub-optimal parameters. Electrophysiological modulation in LC output regions has also been recorded upon VNS (Dorr and Debonnel, 2006; Manta et al., 2009; Alexander et al., 2017; Beaumont et al., 2017). These changes in neuronal activity in LC efferents have also been associated with long-lasting changes in the synaptic proteome in the amygdala and piriform cortex (Alexander et al., 2017). It is worth mentioning that similar studies are absent in the case of the promising non-invasive taVNS in animal models.

Despite Hulsey's rigorous approach toward parameter space exploration, a very rigid set of stimulation parameters is commonly used in animal models as well as in human studies. These involve current intensities varying between 0.25 and 1 mA, pulse frequency ranging between 20 and 30 Hz, a pulse width of 330–500 μs and a duty cycle of 30 s stimulation followed by a 5 min resting phase for 30–60 min (Manta et al., 2009; He et al., 2013b; Jiang et al., 2016; Vázquez-Oliver et al., 2020). Nevertheless, animal research offers the possibility to easily explore new sets of parameters such as variable waveforms or summation effects of multiple stimulation locations (Ay et al., 2016; Kaniusas et al., 2019a). In this respect, biphasic waveforms have been lately proposed since they can lead to larger recruitment of nerve fibers compared to monophasic waveforms (Kaniusas et al., 2019a). Monophasic, biphasic and triphasic stimulation patterns for different bursts lengths were recently compared (Kaniusas et al., 2020). This aspect of being able to manipulate the waveform, therefore, may allow us to tailor the strength of our stimulation depending on the specific disease condition. Furthermore, the majority of reports fail to provide a convincing rationale behind their parameter selection (Hosoi et al., 2000; Huston et al., 2007), stating them as “customized” and thus hindering the optimization of these stimulation parameters (Noller et al., 2019). Given that tVNS finds its application in a range of conditions, just as in the human studies noted above, it will be of prime importance to scrutinize factors such as the stimulation parameters, the anatomical location to deliver the electrical stimulation on the VN, and the design of the electrodes (Noller et al., 2019). Overall, optimization of stimulation parameters derived from animal research may provide an essential basis for optimal tVNS in human patients.

Nevertheless, electrophysiological read-outs might not always be the most suitable output signal to tune stimulation parameters. Even though specific stimulation parameters can evoke robust neuronal spiking, it can also lead to neurotransmitter depletion at the terminals (Yavich et al., 2005). Therefore, higher spiking rates do not necessarily translate into increasing levels of neuromodulators at the extracellular space. Thus, when spike rate is used as the only output optimization variable, the final results can be skewed. In this context, neurochemical approaches became a potent tool that is routinely implemented in animal models but is still far from being applicable in humans. Pioneering studies in the neurochemistry field using microdialysis identified glutamate release in the NTS of cats as a likely mode of vagal neurotransmission (Allchin et al., 1994). Since noradrenergic, cholinergic, or serotonergic activation downstream of the NTS likely mediates therapeutic effects of VNS, synaptic exhaustion can lead to a ceiling of neurotransmitter/neuromodulator levels at lower stimulation frequency as determined by microdialysis (Roosevelt et al., 2006; Follesa et al., 2007; Raedt et al., 2011; Manta et al., 2013). Nevertheless, if real-time feedback is intended for optimization of stimulation parameters using neuromodulator concentration as the output variable, the temporal resolution of microdialysis is too low. Here, electrochemical methods such as cyclic voltammetry and amperometry can be a suitable alternative given their subsecond time resolution (Kile et al., 2012). In particular, neuromodulators such as dopamine, adrenaline, noradrenaline, ATP, and serotonin can be electrochemically detected via fast-scan cyclic voltammetry or amperometry *in vivo* (Heien et al., 2004; John and Jones, 2007; Gourine et al., 2008; Njagi et al., 2010). Thus, fast electrochemical detection of neuromodulator concentration can help to optimize tVNS parameters for a personalized intervention in different pathologies (Mirza et al., 2019). Bringing together both, stimulation optimization and high-speed detection of neuromodulator release, will help to dissect the complex brain state dependence seen in human studies. It is worth noting that using neurotransmitter concentration as an output variable for stimulation parameter optimization can be easily implemented in animal models, with the advantage of multiple recordings in different regions simultaneously and high-density channel recordings (Zhang et al., 2018; Tomagra et al., 2019). Yet, due to its invasive nature, application in humans is precluded, which emphasizes the need for preclinical research on non-human primates.

A unique opportunity in animal research compared to humans will be the dissection of afferent and efferent pathways on a cellular and molecular level. Early retrograde tracing studies have helped us to understand how the auricular branch of the VN innervates brainstem nuclei (Jacquin et al., 1982; Takemura et al., 1987). The auriculotemporal nerve and auricular branch of the VN are thought to predominantly project to the NTS, dorsal vagal nucleus, motor nucleus of the VN, and paratrigeminal nucleus. A picture emerged where most innervation to these nuclei show a strong ipsilateral profile, although the area postrema is a notable exception, as it has bilateral innervation (Kalia and Sullivan, 1982). Despite these pioneering studies, new genetic and viral approaches in animals will continue to unlock the main cellular connectivity pathways involved in tVNS (Nassi et al., 2015). Such connectivity schemes are likely to guide mechanistic approaches to optimally stimulate neuromodulatory systems and better anticipate off-targets effects. It is worthwhile to note that the auricular branch of the VN stimulation zone is innervated by sympathetic nerves as well. It has been therefore suggested that several sympathetic pathways could be stimulated while stimulating the auricular branch of the VN, which might lead to an activation of the NTS via the LC (Cakmak, 2019). This suggestion is novel since unidirectional NTS to LC activation is usually considered (Cakmak, 2019). On the other hand, there is accumulating evidence showing that the LC itself is not a functional neuroanatomical unit, but instead has multiple modules that differ in their projection targets and activity dynamics (Chandler et al., 2019). For example, circuits analysis using viral tracing, optogenetics, and chemogenetics have unraveled specific LC modules/circuits involved in analgesia, explorative behavior, or aversive learning modulation (Hirschberg et al., 2017; Borodovitsyna et al., 2018; Chandler et al., 2019). Moreover, it has been shown that NA is released in the hippocampus after 0.5 mA current stimulation but not in the cortex, while both structures are flooded by the neuromodulator when threshold current crosses more than 1.0 mA (Roosevelt et al., 2006). This exciting finding highlights: (1) the possibility of targeting different networks based on stimulation parameters and (2) the importance of understanding susceptibility of sub-circuits within the noradrenergic system regarding the stimulation parameters as well as different pathologies or brain states.

Animal research will reveal complex neuroanatomical connectivity implicated in taVNS with LC/NTS modularity. As a corollary, researchers will have to keep in mind that parameter optimization should be tuned specifically to the disease or brain state to be modulated, taking into account specific functional neuroanatomy. High throughput recording techniques such as calcium imaging and high-density *in vivo* electrophysiology coupled to molecular genetics and viral tracing will be needed together in this quest (Nassi et al., 2015; Schwarz et al., 2015; Totah et al., 2019). In addition, high-density channel electrochemical methods, behavioral studies and specific transgenic models of disease will also be required to provide a general view on tVNS/taVNS effects at the organismic level during normal conditions and in disease (Zhang et al., 2016, 2018; Tomagra et al., 2019; Vázquez-Oliver et al., 2020).

## Conclusions

Given that the VN has been implicated in the pathophysiology of a number of disorders across many disciplines and phenomena on a behavioral and psychological level, VNS, and particularly non-invasive tVNS, has generated considerable interest. Whilst the mechanisms by which tVNS exerts psychological and physiological effects are increasingly, and more completely, understood, many early studies have been beset by inconsistencies around reporting. The development of internationally agreed consensus guidelines around reporting of tVNS studies should address these issues. Whilst tVNS represents a potential treatment option in many disorders and an interesting tool for experimental research, it needs to be studied in an objective and robust manner before its true place as a neuroimmunomodulatory intervention can be determined.

## Author's Note

If you are interested in being part of the tVNS consensus group and our efforts to strengthen tVNS research methodology, please contact the corresponding author. Finding consensus among so many authors involved is certainly a challenge. We therefore like these recommendations to be considered as work in progress.

## Author Contributions

All authors listed have made a substantial, direct and intellectual contribution to the work, and approved it for publication.

## Conflict of Interest

EK and SK are employed by company SzeleSTIM GmbH. JS received honoraria from SzeleSTIM GmbH and owns patents in the field of the auricular vagus nerve stimulation. EK, SK, and JS are shareholders of SzeleSTIM GmbH. The remaining authors declare that the research was conducted in the absence of any commercial or financial relationships that could be construed as a potential conflict of interest. The reviewer TS declared a shared affiliation, with no collaboration, with one of the authors HJ, to the handling editor at the time of review.

## Footnotes

^1^The review of studies is based on a PubMed search using the keywords “transcutaneous vagus nerve stimulation” OR “tVNS”
